# CRTC1 enhances PD-L1-mediated tumor immunosuppression in non-small cell lung cancer via the Notch1/Akt signaling pathway

**DOI:** 10.3389/fimmu.2025.1658679

**Published:** 2025-09-05

**Authors:** Xujun Feng, Yuan Shi, Fang Yuan, Yanxia Hu, Xiangdong Tang, Wei Zhang, Jiadi Gan, Longhua Sun, Lingling Cao

**Affiliations:** ^1^ The Department of Respiratory and Critical Care Medicine, The First Affiliated Hospital, Jiangxi Medical College, Nanchang University, Nanchang, Jiangxi, China; ^2^ Jiujiang City Key Laboratory of Cell Therapy, The First Hospital of Jiujiang City, Jiujiang, Jiangxi, China; ^3^ Sleep Medicine Center, Department of Respiratory and Critical Care Medicine, Mental Health Center, West China Hospital, Sichuan University, Chengdu, Sichuan, China; ^4^ Department of Respiratory, The First Hospital of Jiujiang City, Jiujiang, Jiangxi, China; ^5^ Jiangxi Provincial Key Laboratory of Respiratory Diseases, The First Affiliated Hospital, Jiangxi Medical College, Nanchang University, Nanchang, Jiangxi, China; ^6^ Department of Pulmonary and Critical Care Medicine, State Key Laboratory of Respiratory Health and Multimorbidity, West China Hospital, West China School of Medicine, Sichuan University, Chengdu, Sichuan, China

**Keywords:** non-small cell lung cancer, immunotherapy, immunosuppression, CRTC, Notch/Akt signaling

## Abstract

**Background:**

While programmed death-ligand 1 (PD-L1)-targeted immunotherapy represents an advancement in non-small cell lung cancer (NSCLC), patient outcomes remain suboptimal. Aberrant activation of the cyclic adenosine monophosphate (cAMP) response element binding protein (CREB)-regulated transcription coactivator (CRTC) is linked to malignant proliferation and functionality in lung cancer cells. This study investigates the involvement of CRTC1 in tumor immunity.

**Methods:**

CRTC1 and Notch1 expression were regulated in A549 and NCI-H1299 NSCLC lines through plasmid-mediated overexpression/silencing to assess their effects on cell viability, apoptosis, migration, and invasion. CRTC1/Notch1-dysregulated Lewis lung carcinoma (LLC) cells were co-cultured with T cells to evaluate T cell activation and function. The efficacy of combined CRTC1 knockdown/overexpression and atezolizumab (anti-PD-L1) was tested in an LLC xenograft mouse model.

**Results:**

CRTC1 promoted cell viability, migration, and invasion while suppressing apoptosis across NSCLC models. In LLC cells, CRTC1 upregulated tumor cell PD-L1 expression, suppressed T cell-derived IFN-γ and IL-2 production, diminished endogenous CXCL10/11 secretion, and impaired T cell proliferation and cytotoxicity. Mechanistically, CRTC1 interacted with Notch1 to activate the Notch1/Akt pathway, stimulating PD-L1 upregulation, thereby facilitating tumor immunosuppression and growth. Notably, CRTC1 overexpression reversed the protective effects of atezolizumab on tumor growth. Combining CRTC1 knockdown with atezolizumab synergistically enhanced anti-tumor T cell immunity, achieving the most significant tumor regression in xenografts.

**Conclusion:**

These findings indicate that CRTC1 in tumor cells suppresses PD-L1-mediated anti-tumor immunity and promotes tumorigenesis via the Notch1/Akt signaling axis. Dual targeting of CRTC1 and PD-L1 demonstrates therapeutic synergy, suggesting CRTC1 pathway inhibition could optimize immunotherapy outcomes in NSCLC patients.

## 1 Introduction

Lung cancer persists as the leading global cause of cancer-related morbidity and mortality (1). Non-small cell lung cancer (NSCLC), accounting for approximately 85% of pulmonary malignancies, demonstrates a poor 5-year survival prognosis (<20%) despite therapeutic advances (2). Early-stage NSCLC (stages I-II) is primarily managed with curative-intent surgical resection, supplemented with platinum-based chemotherapy. Chemoradiotherapy is widely used for non-surgical candidates (stages III-IV) (3). Notably, 60%-70% of patients are diagnosed at advanced stages (4), where chemoradiotherapy offers limited survival benefits for locally advanced or metastatic disease (5). The advent of immune checkpoint inhibitors (ICIs) targeting programmed death 1 (PD-1)/PD-L1 axis has transformed therapeutic paradigms, with agents like atezolizumab achieving first-line approval for advanced NSCLC (6). However, only 20-30% of patients respond to monotherapy, and challenges including resistance and toxicities persist (7). Emerging evidence suggests combinatorial approaches may overcome these limitations by augmenting T cell-mediated antitumor responses and prolonging survival benefits (8).

The PD-1/PD-L1 checkpoint axis serves as a critical immunosuppressive pathway in oncology, enabling malignant cells to evade T cell-mediated cytotoxicity by subverting lymphocyte activation and effector functions (9). CD274-encoded PD-L1, frequently overexpressed across solid tumors, engages PD-1 receptors on infiltrating T lymphocytes to downregulate proximal TCR kinases, induce lymphocyte apoptotic pathways, and attenuate effector cytokine production (10). This ligand-receptor interplay establishes a fundamental immune resistance mechanism underlying tumor immune escape and disease progression. Modulating PD-L1 expression in NSCLC cells can increase susceptibility to T cell cytotoxicity, thereby influencing cancer cell survival, proliferation, and migration (11).

PD-L1-mediated immune escape and tumor growth are dynamically regulated by oncogenic factors (12). CREB-regulated transcription co-activators (CRTCs) undergo dephosphorylation and nuclear translocation in response to cyclic-AMP (cAMP), where they bind cAMP response element-binding protein (CREB) to regulate diverse cellular processes (13). The CRTC family comprises three members (CRTC1-3). Aberrant CRTC signaling has been linked to aggressive phenotypes in lung malignancies (14). Prior studies report that CRTC2 activation upregulates LINC00963 (MetaLnc9) to drive NSCLC cell migration and invasion (15). CRTC2 has also been shown to downregulate PD-1/PD-L1, and its knockdown reverses primary resistance to anti-PD-1 therapy in hepatocellular carcinoma (16). However, the direct role of CRTCs in regulating PD-L1 expression and immunotherapy efficacy in NSCLC remains unexplored.

The Notch pathway, an evolutionarily conserved signaling cascade, plays critical roles in NSCLC metabolic reprogramming and tumor microenvironment regulation (17). Notch1 hyperactivation in NSCLC enhances malignant phenotypes and chemoresistance (18, 19) and promotes immune evasion via PD-L1 upregulation (20). Protein kinase B (Akt), a key downstream effector of Notch signaling, cooperatively regulates tumor growth and metastasis (21). Akt phosphorylation also positively correlates with PD-L1 activation in NSCLC cells (22), suggesting Notch/Akt signaling may modulate PD-L1-mediated T cell dysfunction. Whether CRTCs influence this process remains unknown.

In this study, we employed atezolizumab, an anti-PD-L1 monoclonal antibody, to investigate the therapeutic impact of combining CRTC1 modulation with PD-L1 blockade in NSCLC xenografts. *In vivo* data revealed that CRTC1 overexpression attenuated atezolizumab efficacy, whereas CRTC1 knockdown synergistically enhanced tumor suppression. *In vitro*, CRTC1 overexpression promoted tumor cell growth, migration, invasion, and T cell immunosuppression. Mechanistically, CRTC1 interacted with Notch1 to activate Notch/Akt signaling, driving PD-L1 expression and suppressing pro-inflammatory/chemokine release. Our data demonstrate that CRTC1-targeted intervention constitutes a viable strategy to potentiate anti-PD-L1 therapy for NSCLC.

## 2 Methods

### 2.1 Bioinformatics analysis

Publicly available human NSCLC datasets, including 482 tumor samples and 109 adjacent normal tissues, were obtained from The Cancer Genome Atlas (TCGA). Immune cell infiltration scores in the tumor microenvironment were calculated using the MCP-counter algorithm. A heatmap was generated to visualize Pearson correlations between CRTC1 expression levels and immune cell infiltration scores.

### 2.2 Cell culture and treatment

Human NSCLC cell lines (A549 [AW-CCH011] and NCI-H1299 [AW-CCH038]), human normal lung epithelial cells (BEAS-2B; AW-CNH004), mouse Lewis lung carcinoma (LLC) cells (AW-CCM076), mouse normal lung epithelial cells (MLE-12; AW-CNM486), and mouse T cells (CTLL-2; AW-CCM556) were procured from Abiowell (Changsha, China). A549 cells were cultured in F-12 K medium, NCI-H1299 in RPMI-1640, and MLE-12/LLC cells in DMEM. CTLL-2 cells were cultured in RPMI-1640 supplemented with 100 U/mL recombinant mouse interleukin-2 (rmIL-2) and 1 μg/mL concanavalin A (Con A). All media were supplemented with 10% fetal bovine serum (FBS) and 1% penicillin/streptomycin, with cells maintained at 37 °C under 5% CO_2_.

To investigate CRTC1/Notch1 roles, A549 and H1299 cells were transfected for 48 h with plasmids overexpressing CRTC1 (oe-CRTC1), knocking down CRTC1 (si-CRTC1), overexpressing Notch1 (oe-Notch1), or negative control vectors (oe-NC or si-NC; HonorGene, Changsha, China) using Lipofectamine 2000 reagent (11668019, Thermo Fisher Scientific, Waltham, MA, USA).

### 2.3 Co-culture and T cell-mediated tumor cell killing assay

LLC cells (5 × 10^4^/well) were seeded in 24-well plates and transfected with oe-CRTC1, si-CRTC1, si-CRTC1 + oe-Notch1, or control vectors for 48 h, followed by medium replacement with fresh 10% FBS-containing medium. CTLL-2 T cells were pre-activated with 100 U/mL IL-2 for 24 h and co-cultured with LLC cells (5 × 10^5^/well) for 24 h at 37 °C under 5% CO_2_ (23, 24). Supernatants were collected for lactate dehydrogenase (LDH) release quantification (A020-2-2, Nanjing Jiancheng Bioengineering Institute, Nanjing, China). Cytotoxicity was calculated as: Cytotoxicity (%) = (Experimental LDH release – Spontaneous LDH release)/(Maximum LDH release – Spontaneous LDH release) × 100.

### 2.4 Animals and xenograft tumor model

Male C57BL/6J mice (5–6 weeks old) were purchased from Hunan SJA Laboratory Animal Co., Ltd. Animal experiments were approved by the Animal Ethics Committee of Jiujiang First Hospital (No. JJSDYRMYY-YXLL-2024-064) and followed the NIH Guide for the Care and Use of Laboratory Animals. Mice were subcutaneously injected with 100 μL LLC cells (1 × 10^6^) into the right flank (25). Atezolizumab (5 mg/kg; HY-P9904, MCE, Monmouth Junction, NJ, USA) was administered intraperitoneally every 5 days starting from day 7 post-inoculation.

To assess CRTC1 overexpression, mice were implanted with oe-CRTC1-transfected LLC cells (1 × 10^6^) and treated with atezolizumab, while control mice received oe-NC-transfected cells (*n* = 5). For CRTC1/Notch1 functional studies, mice were grouped to receive LLC cells transfected with si-NC, si-CRTC1, si-CRTC1 + oe-NC, or si-CRTC1 + oe-Notch1, followed by atezolizumab treatment (*n* = 5). Tumor volume was measured every 3 days as 0.5 × length × width². On day 18, mice were euthanized via sodium pentobarbital overdose, and tumors were excised and weighed.

### 2.5 Hematoxylin and eosin staining

Tumor samples were fixed in 4% paraformaldehyde, paraffin-embedded, and sectioned at 5 μm. Deparaffinized sections were rehydrated through graded ethanol, stained with hematoxylin and eosin (AWI0020, Abiowell), dehydrated in ascending ethanol, cleared in xylene, and mounted with neutral resin. Tissue morphology was examined under a light microscope (BA210T, Motic, Xiamen, China).

### 2.6 Immunohistochemical staining

Deparaffinized tumor sections underwent antigen retrieval in citrate buffer (pH 6.0) via microwave heating. Endogenous peroxidase activity was blocked with 1% periodic acid for 15 min. Sections were incubated overnight at 4 °C with anti-PD-L1 (1:300; ab213524, Abcam, Cambridge, UK), followed by phosphate-buffered saline (PBS) washing and incubation with horseradish peroxidase (HRP)-conjugated secondary antibody (1:200; AWI0629, Abiowell) at room temperature for 1 h. Diaminobenzidine (DAB) was used for chromogenic detection, with hematoxylin counterstaining. Images were analyzed using Image-Pro-Plus software (Media Cybernetics, Rockville, MD, USA).

### 2.7 Quantitative real-time PCR

Total RNA was isolated from cells using TRIzol reagent (15596026, Thermo Fisher Scientific), and cDNA was synthesized with the HiFiScript cDNA Synthesis Kit (CW2569M, CWBIO, Taizhou, China). *CRTC1* mRNA levels were quantified on a QuantStudio1 Real-Time PCR System (Thermo Scientific) using UltraSYBR Mixture (CW2601M, CWBIO). Relative gene expression was calculated via the 2^−ΔΔCt^ method normalized to *β-actin*. Primers included: Human CRTC1: 5’-CTTCCAGCCCAGCGGATTTCT-3’ (forward) and 5’-AGGATTGGAAGGGGGTCAGAG-3’ (reverse) and β-actin: 5’-ACCCTGAAGTACCCCATCGAG-3’ (forward) and 5’-AGCACAGCCTGGATAGCAAC-3’ (reverse). All reactions were performed in triplicate.

### 2.8 Western blot analysis

Proteins were extracted from cells and tumors using RIPA lysis buffer (R0010, Solarbio, Beijing, China), separated by SDS-PAGE, and transferred to nitrocellulose membranes. Membranes were blocked in Tris-buffered saline containing 0.2% Tween-20 and 5% skim milk, then incubated overnight at 4 °C with primary antibodies. After PBS washing, membranes were incubated with HRP-conjugated secondary antibodies (Proteintech, Rosemont, IL, USA) for 1 h at room temperature. Signals were visualized using enhanced chemiluminescence (ECL) on a ChemiScope6100 system (CLiNX, Shanghai, China). Densitometric analysis was performed with ImageJ software (NIH, Bethesda, MD, USA), with β-actin as the loading control. Antibodies included: CRTC1 (1:1000; AWA03132, Abiowell), PD-L1 (1:1000; 28076-1-AP, Proteintech), Notch1 (1:2000; 10062-2-AP, Proteintech), phospho-Akt (Ser473) (p-Akt; 1:5000; 66444-1-Ig, Proteintech), Akt (1:5000; 60203-2-Ig, Proteintech), CXCL10 (1:2000; 10937-1-AP, Proteintech), CXCL11 (1:1000; 10707-1-AP, Proteintech), and β-actin (1:5000; 66009-1-Ig, Proteintech).

### 2.9 Co-immunoprecipitation assay

Cells were lysed in IP lysis buffer, sonicated, and incubated on ice for 30 min, followed by centrifugation at 12,000 rpm for 10 min at 4°C. Cell lysates were incubated with anti-CRTC1, anti-Notch1 antibodies, or control IgG (Proteintech) under rotation overnight at 4°C. The immunoprecipitates were conjugated with protein A/G agarose beads at 4°C for 2h. After repeated washing with IP lysis buffer, the beads were subjected to Western blot analysis using anti-CRTC1 and anti-Notch1 antibodies.

### 2.10 Cell viability assay

Cell viability was assessed using the CCK-8 assay (Dojindo Laboratories, Tokyo, Japan). Cells (5 × 10³/well) were seeded in 24-well plates. After 24h, 10% CCK-8 solution was added to each well. Following 4h of incubation, absorbance at 450 nm was measured using a microplate reader.

### 2.11 Transwell assay

Cells (2 × 10^6^/mL, 500 μL) in serum-free medium were seeded onto Matrigel-coated (200 μg) Transwell inserts. The lower chamber of the 6-well plate contained complete medium with 10% FBS. After 48h incubation, inserts were fixed with 4% paraformaldehyde, stained with 0.1% crystal violet, and migrated cells were quantified by averaging counts from three random fields.

### 2.12 Wound healing assay

Confluent cell monolayers in 6-well plates were scratched vertically using a pipette tip. After PBS washing, cells were cultured in serum-free medium at 37°C. Wound closure was photographed at 0, 24, and 48h.

### 2.13 Apoptosis assay

Cells (1 × 10^5^) in 6-well plates were stained using an Annexin V-APC/PI Apoptosis Kit (KGA1030, KeyGen BioTECH, Nanjing, China). Flow cytometry was performed within 1h on a CytoFLEX analyzer (Beckman Coulter, Fullerton, CA, USA).

### 2.14 ELISA

CXCL10 (CSB-E08183m), CXCL11 (CSB-EL006241MO), IFN-γ (CSB-E04578m), and IL-2 (CSB-E04627m) levels in cell supernatants and serum were measured using commercial kits (CUSABIO, Wuhan, China) according to the manufacturer’s instructions. Optical density values were determined with a microplate reader.

### 2.15 Statistical analysis

Data are expressed as mean ± standard deviation (SD). The Shapiro-Wilk test was employed to check normality, whereas the Brown-Forsythe test was utilized to evaluate homoscedasticity in GraphPad Prism. Differences between two groups were assessed using Student’s *t*-test, while multi-group comparisons utilized ANOVA with Tukey’s *post-hoc* test. Pearson correlation coefficient was employed to evaluate the correlation between CRTC1 and Notch1, PD-L1, as well as CXCL10 and CXCL11. A p-value < 0.05 was considered statistically significant.

## 3 Results

### 3.1 CRTC1 compromises PD-L1 checkpoint efficacy in NSCLC tumor elimination

**Figure 1:**
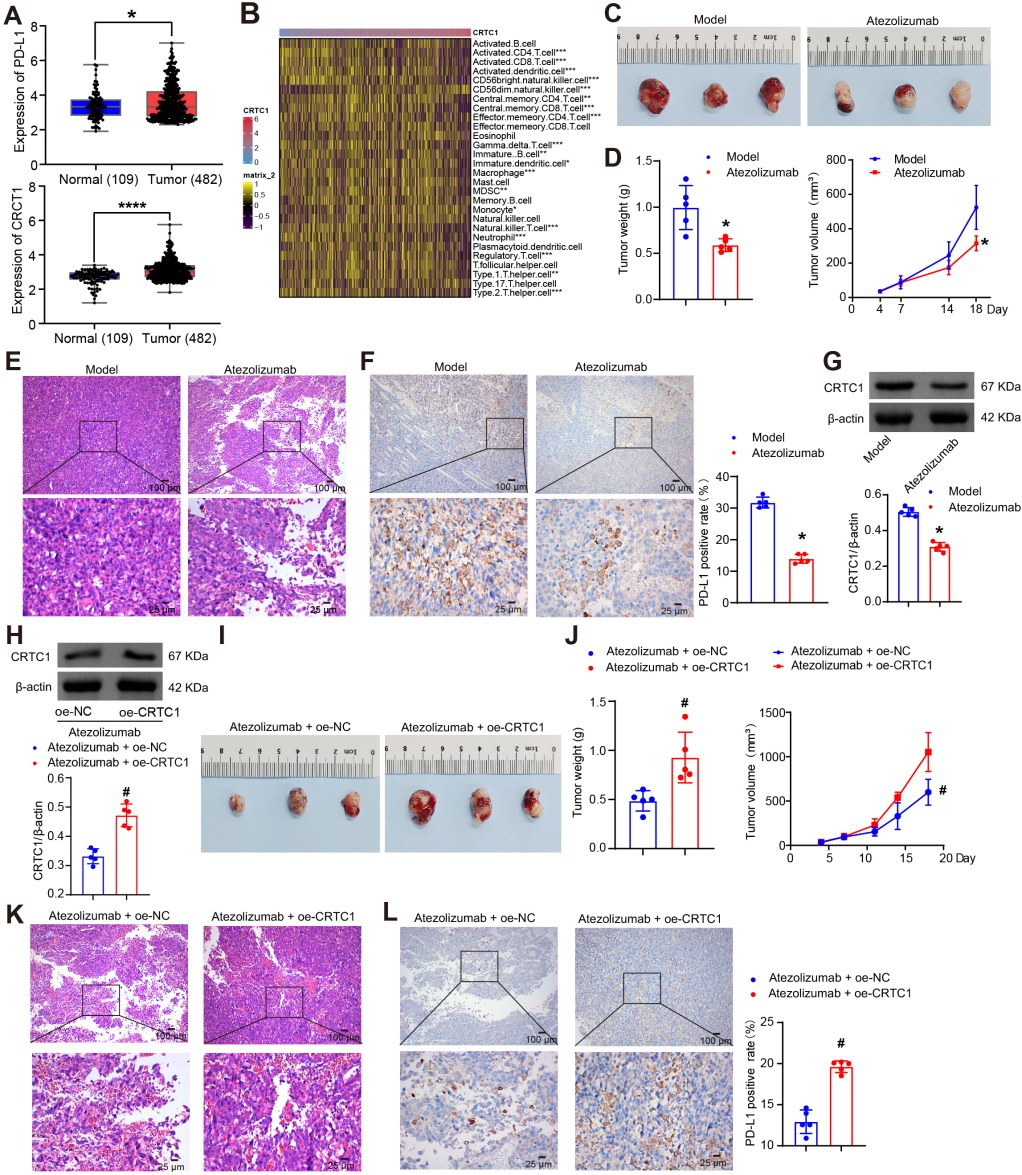
CRTC1 impairs the therapeutic efficacy of anti-PD-L1 therapy in NSCLC tumors. **(A)** Box plots showing CRTC1 and CD274 (encoding PD-L1) expressions in NSCLC tumor samples (*n* = 482) and adjacent normal tissues (*n* = 109) from the TCGA database. *p < 0.05, ****p < 0.0001 vs. Normal. **(B)** Heatmap illustrating correlations between CRTC1 and immune cell infiltration levels. *p < 0.05, **p < 0.01, ***p < 0.001. **(C)** Tumor size in C57BL/6J mice bearing Lewis subcutaneous xenograft tumors after Atezolizumab treatment. **(D)** Tumor volume and weight of Lewis xenograft tumors after Atezolizumab treatment. **(E)** Representative H&E staining images of Lewis xenograft tumors after Atezolizumab treatment. Scale bar = 100 μm (top) and 25 μm (bottom). **(F)** IHC analysis of PD-L1 expression in Lewis xenograft tumors after Atezolizumab treatment. Scale bar = 100 μm (top) and 25 μm (bottom). **(G)** Western blot analysis of CRTC1 expression in Lewis xenograft tumors after Atezolizumab treatment. **(H)** Western blot validation of CRTC1 overexpression efficiency in Lewis xenograft tumors treated with atezolizumab and plasmid overexpressing CRTC1 (oe-CRTC1) or its negative control (oe-NC). **(I)** Tumor size in C57BL/6 mice bearing Lewis xenograft tumors with CRTC1 overexpression combined with Atezolizumab treatment. **(J)** Tumor volume and weight of Lewis xenograft tumors in mice receiving CRTC1 overexpression and Atezolizumab. **(K)** Representative H&E staining images of Lewis xenograft tumors under combined CRTC1 overexpression and Atezolizumab treatment. Scale bar = 100 μm (top) and 25 μm (bottom). **(L)** IHC analysis of PD-L1 expression in Lewis xenograft tumors treated with CRTC1 overexpression and Atezolizumab. Scale bar = 100 μm (top) and 25 μm (bottom). *n* = 5. The p-values in **(D, F, G, H, J, L)** were calculated using Student’s *t*-test. *p < 0.05 vs. Model. #p < 0.05 vs. Atezolizumab + oe-NC.

Interrogation of TCGA datasets revealed concurrent upregulation of CRTC1 and PD-L1 (*CD274*) in NSCLC tumor specimens (**Figure 1A**). CRTC1 expression was correlated strongly with immune cell infiltration, particularly T cells (**Figure 1B**). Given that PD-L1 blockade reactivates exhausted T cells to restore antitumor immunity (26), we investigated whether CRTC1 modulates anti-PD-L1 therapy response. C57BL/6J mice bearing established LLC xenografts (day 7 post-implantation) were randomized into control and treatment groups. The treatment group received daily intraperitoneal injections of 5 mg/kg atezolizumab. Ten days after treatment initiation, atezolizumab significantly reduced tumor size, weight, and volume (**Figures 1C–D**). H&E staining of LLC tumors revealed enhanced tumor cell death (**Figure 1E**), while IHC and Western blot confirmed PD-L1 downregulation (**Figure 1F**) and reduced CRTC1 protein levels (**Figure 1G**) in treated tumors, respectively. Under atezolizumab treatment, CRTC1 overexpression in LLC tumors accelerated tumor growth, reduced tumor cell death (**Figures 1H–K**), and upregulated PD-L1 (**Figure 1L**). These results demonstrate that CRTC1 is associated with NSCLC resistance to anti-PD-L1 therapy.

### 3.2 CRTC1 knockdown suppresses tumor cell growth and downregulates PD-L1 *in vitro*

**Figure 2:**
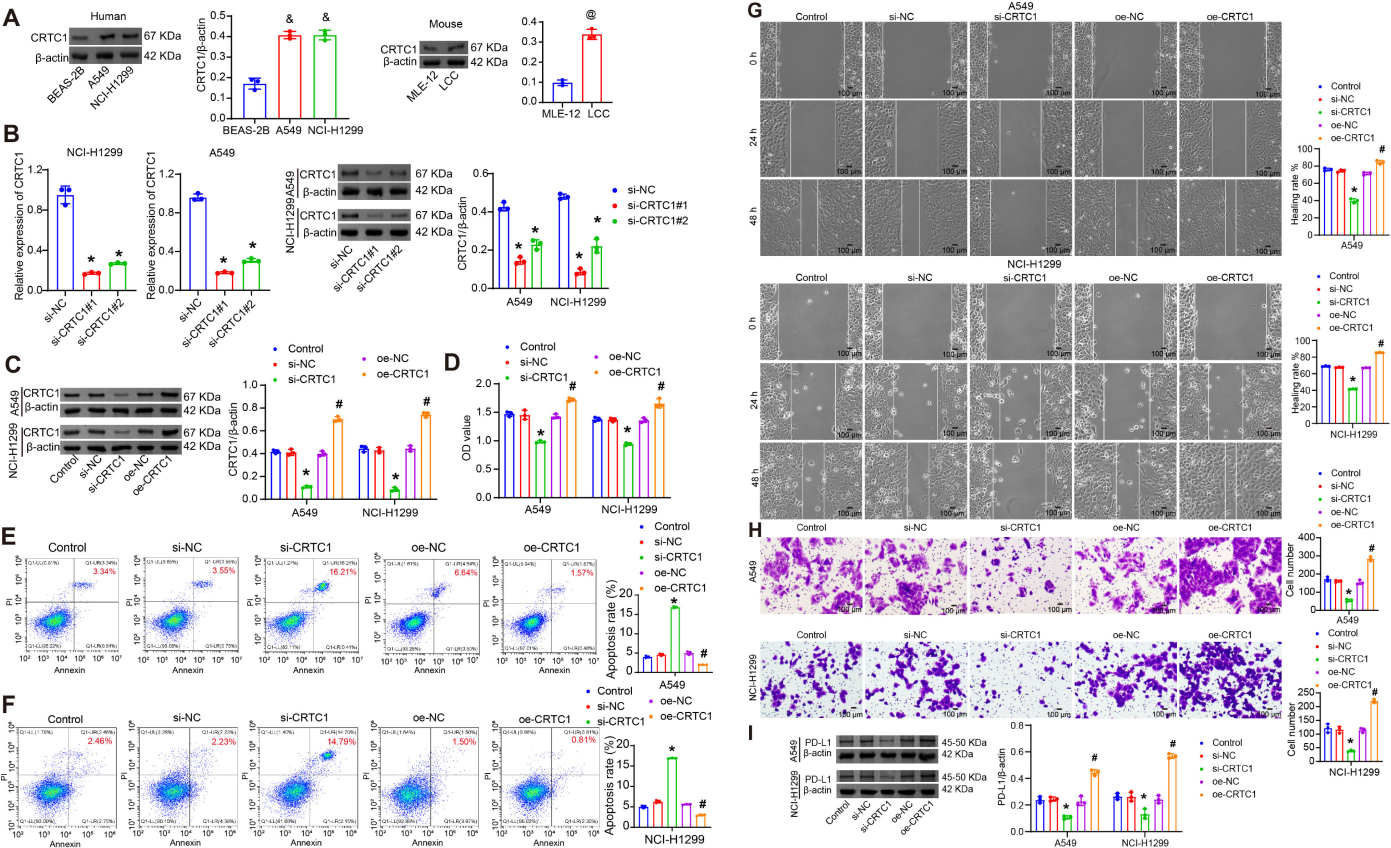
CRTC1 promotes tumor cell growth and PD-L1 expression *in vitro*. **(A)** Western blot analysis of CRTC1 expression in BEAS-2B, A549, NCI-H1299, and LLC cells. **(B)** qRT-PCR and Western blot validation of transfection efficiency for plasmids knocking down CRTC1 (si-CRTC1#1-2) in A549 and NCI-H1299 cells. **(C)** Western blot confirming transfection efficiency of si-CRTC1#1 and plasmid overexpressing CRTC1 (oe-CRTC1) in A549 and NCI-H1299 cells compared to negative controls (si-NC or oe-NC). **(D)** CCK-8 assay assessing cell viability in A549 and NCI-H1299 cells. **(E–G)** Flow cytometry analysis of apoptosis in A549 and NCI-H1299 cells. **(H)** Representative wound closure images at 0, 24, and 48 h showing migration of A549 and NCI-H1299 cells. Scale bar = 100 μm. **(I)** Representative Transwell images showing invasion of A549 and NCI-H1299 cells. Scale bar = 100 μm. **(J)** Western blot analysis of PD-L1 expression in A549 and NCI-H1299 cells. *n* = 3. The p-values in **(A–J)** were calculated using ANOVA with Tukey’s *post hoc* test. *p < 0.05 vs. si-NC. #p < 0.05 vs. oe-NC. &p < 0.05 vs. BEAS-2B. @p < 0.05 vs. MLE-12.

The biological functions of CRTC1 in NSCLC cells were investigated *in vitro*. Compared to BEAS-2B cells, CRTC1 expression was upregulated in A549 and NCI-H1299 cells, and similarly elevated in LLC cells relative to MLE-12 (**Figure 2A**). Transfection with plasmid knocking down CRTC1 (si-CRTC1#1-2) effectively knocked down CRTC1 in A549 and NCI-H1299 cells, with si-CRTC1#1 selected for subsequent experiments, while plasmid overexpressing CRTC1 (oe-CRTC1) increased its expression (**Figures 2B–C**). CRTC1 knockdown suppressed cell viability, migration, and invasion capacities while promoting apoptosis, whereas CRTC1 overexpression produced opposing effects (**Figures 2D–I**). PD-L1 expression was downregulated following CRTC1 knockdown but upregulated upon CRTC1 overexpression (**Figure 2J**). These data collectively demonstrate that CRTC1 promotes tumor cell growth and PD-L1 expression *in vitro*.

### 3.3 CRTC1 targets the Notch1/Akt pathway to drive tumor growth and PD-L1 upregulation

**Figure 3:**
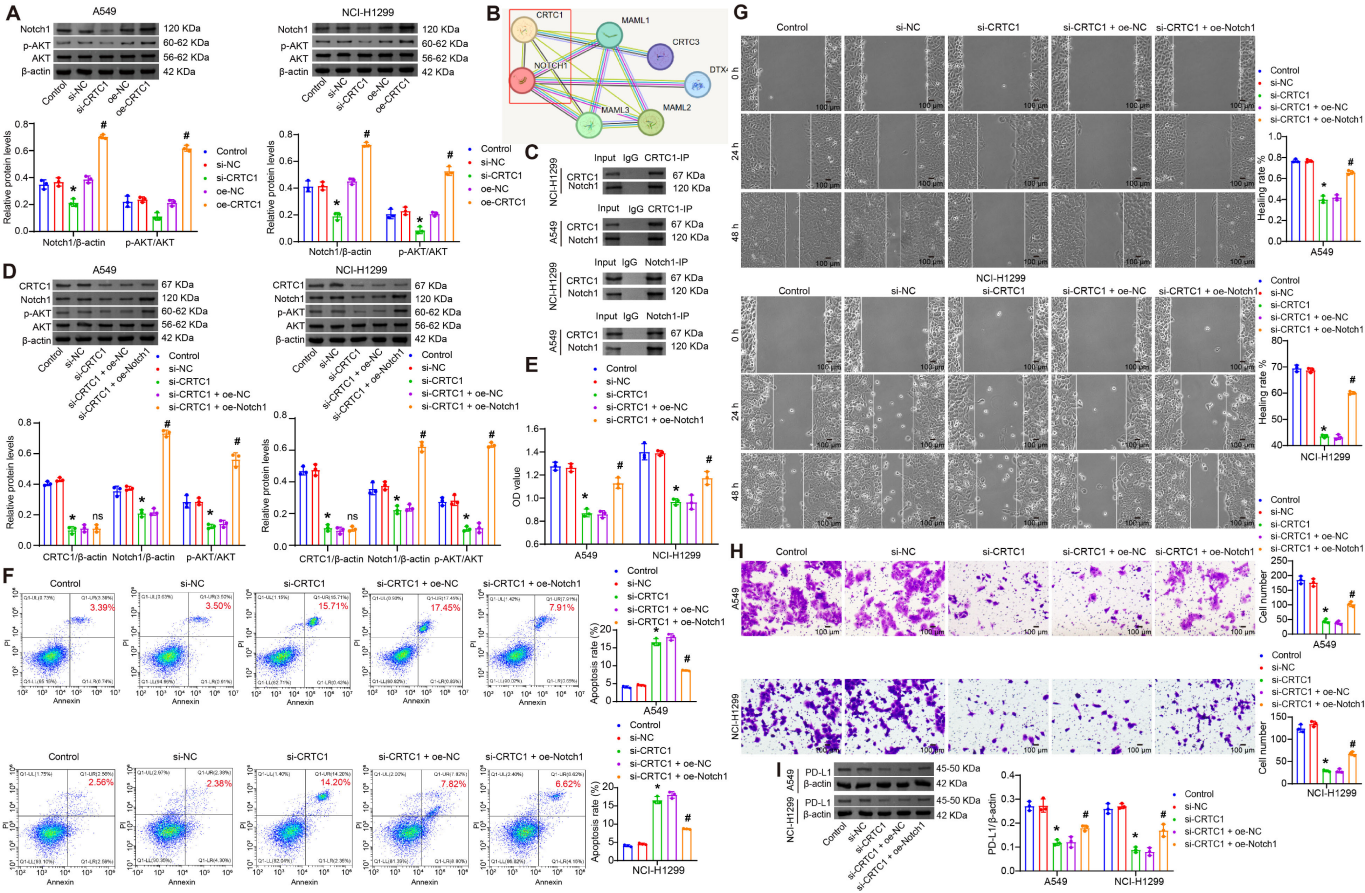
CRTC1 drives tumor cell growth via the Notch1/Akt signaling pathway *in vitro*. **(A)** Western blot analysis of Notch1, Akt, and p-Akt levels in A549 and NCI-H1299 cells transfected with plasmids knocking down CRTC1 (si-CRTC1) or overexpressing CRTC1 (oe-CRTC1) compared to negative controls (si-NC or oe-NC). *n* = 3. *p < 0.05 vs. si-NC. #p < 0.05 vs. oe-NC. **(B)** STRING database-predicted interaction between CRTC1 and Notch1. **(C)** Co-IP validation of CRTC1 and Notch1 interaction in A549 and NCI-H1299 cells. **(D)** Western blot analysis of Notch1, Akt, and p-Akt levels in A549 and NCI-H1299 cells transfected with si-CRTC1 and plasmid overexpressing Notch1 (oe-Notch1). **(E)** CCK-8 assay evaluating cell viability in A549 and NCI-H1299 cells. **(F)** Flow cytometry analysis of apoptosis in A549 and NCI-H1299 cells. **(G)** Representative wound closure images at 0, 24, and 48 h showing migration of A549 and NCI-H1299 cells. Scale bar = 100 μm. **(H)** Representative Transwell images showing invasion of A549 and NCI-H1299 cells. Scale bar = 100 μm. **(I)** Western blot analysis of PD-L1 expression in A549 and NCI-H1299 cells. *n* = 3. The p-values in panels **(A, D–I)** were calculated using ANOVA with Tukey’s *post hoc* test. *p < 0.05 vs. si-NC. #p < 0.05 vs. si-CRTC1 + oe-NC.

Given the established roles of Notch1 in NSCLC proliferation, migration, and tumor immunity (27, 28), along with its capacity to induce Akt phosphorylation (p-Akt) (21), we investigated the involvement of the Notch1/Akt pathway in CRTC1-mediated regulation. In A549 and NCI-H1299 cells, CRTC1 knockdown reduced the levels of Notch1 and the p-Akt/total Akt ratio, whereas CRTC1 overexpression elevated these levels (**Figure 3A**). The STRING database predicted a physical interaction between CRTC1 and Notch1 (**Figure 3B**), which was experimentally validated through Co-IP assays in NSCLC cells (**Figure 3C**). Notch1 overexpression rescued the inhibitory effects of CRTC1 knockdown on Notch1 and p-Akt expression without affecting CRTC1 expression itself (**Figure 3D**). Functionally, Notch1 overexpression restored cell viability, migration, and invasion capacities while suppressing apoptosis (**Figures 3E–H**). The downregulation of PD-L1 induced by CRTC1 knockdown was reversed upon Notch1 overexpression (**Figure 3I**). Collectively, these data demonstrate that CRTC1 promotes tumor growth and PD-L1 expression *in vitro* through the Notch1/Akt pathway.

### 3.4 CRTC1 activation in tumor cells compromises T cell survival and cytotoxicity

**Figure 4:**
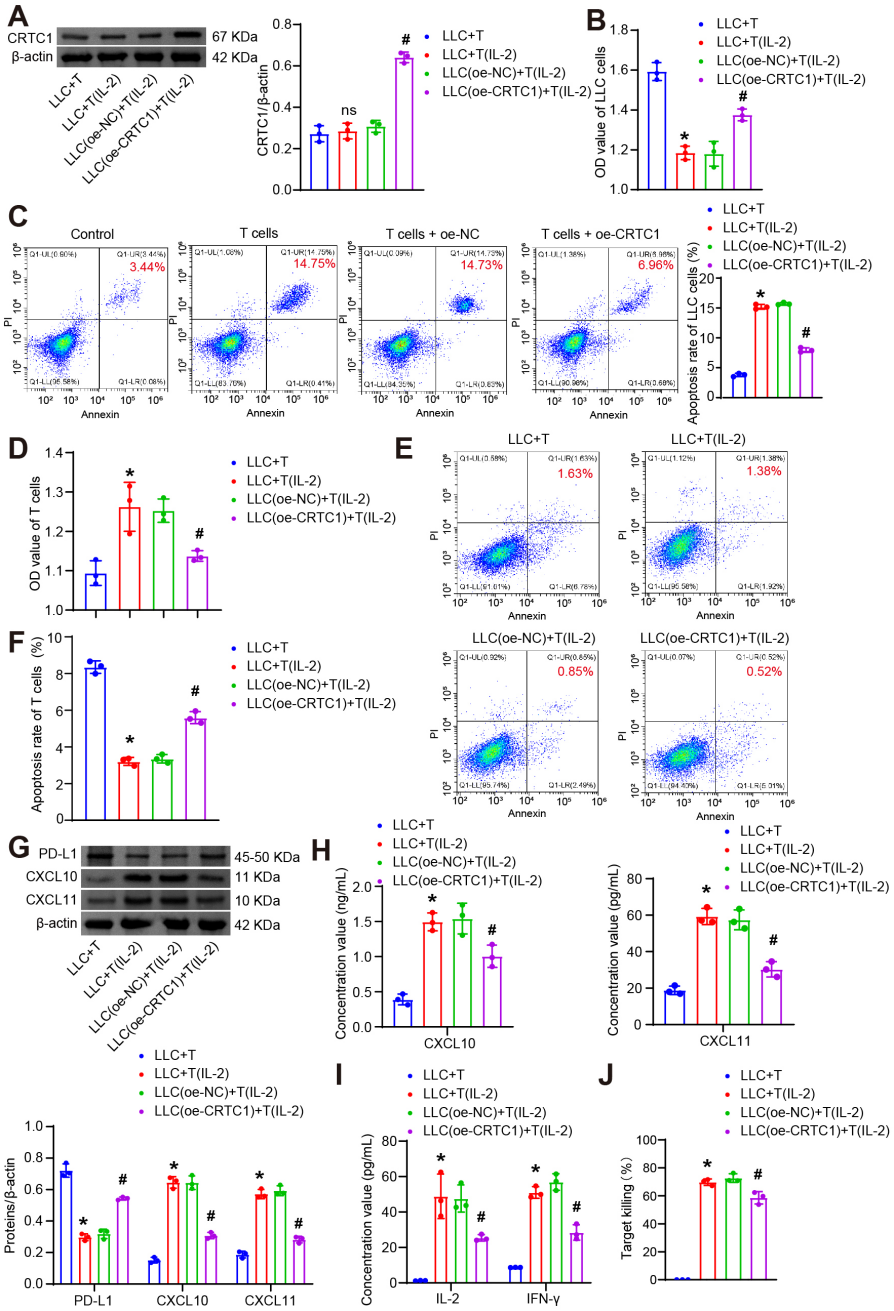
CRTC1 in LLC cells suppresses T-cell proliferation and cytotoxicity. **(A)** Western blot analysis of CRTC1 expression in LLC cells transfected with plasmid overexpressing (oe-CRTC1) or its negative control (oe-NC) and co-cultured with activated CTLL-2 cells at a 1:10 ratio for 24 h **(B)** CCK-8 assay measuring viability of LLC cells. **(C)** Flow cytometry analysis of apoptosis in LLC cells. **(D)** CCK-8 assay assessing T-cell viability. **(E-F)** Flow cytometry analysis of apoptosis in T cells. **(G)** Western blot analysis of PD-L1, CXCL10, and CXCL11 expression in co-cultures. **(H)** ELISA detecting CXCL10 and CXCL11 secretion in supernatants. **(I)** ELISA measuring IFN-γ and IL-2 levels in supernatants. **(J)** LDH release assay evaluating T-cell cytotoxicity via supernatant LDH concentration. *n* = 3. The p-values in panels **(A–J)** were calculated using ANOVA with Tukey’s *post hoc* test. *p < 0.05 vs. Control. #p < 0.05 vs. T cells + oe-NC.

Given the critical role of T cell-mediated immunosuppression in NSCLC tumorigenesis, we established a tumor-T cell co-culture system to evaluate how tumor cell CRTC1 modulates T cell survival and cytotoxic function. In this system, LLC cells transfected with oe-NC or oe-CRTC1 were co-cultured with CTLL-2 T cells either unstimulated or activated with 100 U/mL IL-2. As a T-cell growth factor, IL-2-activated T cells exert dual-directional modulation on the co-culture system (29). IL-2 promotes T-cell proliferation and differentiation. Conversely, IL-2 potentiates antitumor effector functions of T cells, thereby influencing tumor cell viability and functionality. Compared to co-culture with unstimulated T cells (LLC+T group), IL-2-activated T cells did not alter CRTC1 expression in LLC lysates but significantly upregulated it following oe-CRTC1 transfection (**Figure 4A**). IL-2-activated T cells reduced LLC viability and increased apoptosis. Notably, when co-cultured with activated T cells, CRTC1-overexpressing LLC cells exhibited significantly increased LLC viability and reduced apoptosis (**Figures 4B–C**). Conversely, IL-2 stimulation enhanced T cell viability and reduced apoptosis, effects that were reversed by co-culture with CRTC1-overexpressing LLC cells (**Figures 4D–F**). Activated T cells downregulated PD-L1 and upregulated CXCL10/CXCL11 in LLC lysates, which were reversed by CRTC1 overexpression (**Figure 4G**). Supernatant analysis revealed reduced secretion of IFN-γ, IL-2, CXCL10, and CXCL11 upon CRTC1 overexpression (**Figures 4H–I**), indicating impaired T cell activation. Assessment of cytotoxic function via LDH release assay demonstrated that IL-2 activation increased supernatant LDH levels, while CRTC1 overexpression significantly reduced them (**Figure 4J**). Collectively, these data demonstrate that CRTC1 in LLC cells impairs T cell function.

### 3.5 CRTC1 attenuates T cell survival and cytotoxicity via the Notch1/Akt pathway

**Figure 5:**
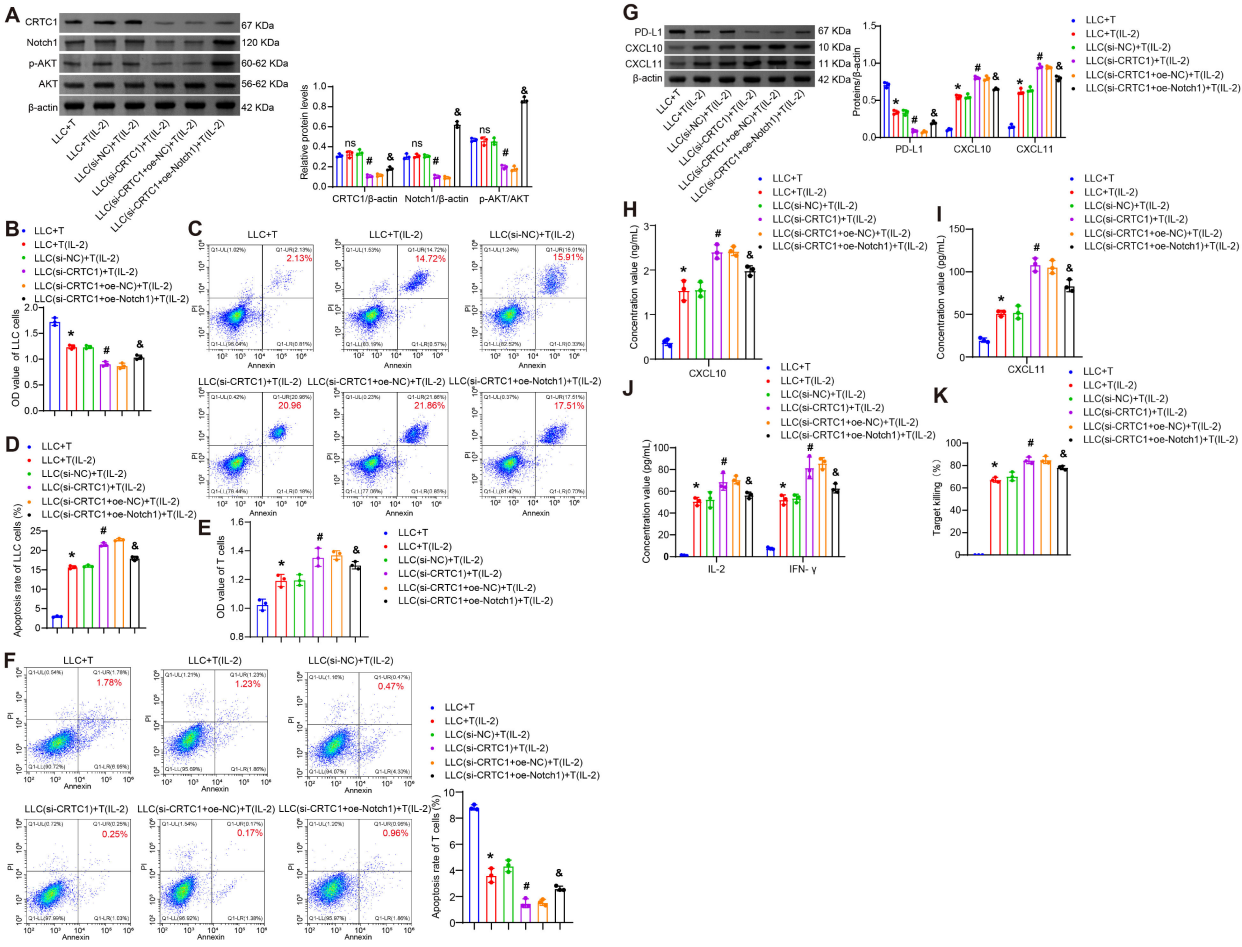
CRTC1 in LLC cells inhibits T-cell proliferation and cytotoxicity via Notch1/Akt. **(A)** Western blot analysis of CRTC1, Notch1, and p-Akt expression in LLC cells transfected with plasmids knocking down CRTC1 (si-CRTC1) and overexpressing Notch1 (oe-Notch1) or their negative controls (si-NC or oe-NC), followed by co-culture with activated CTLL-2 cells (1:10 ratio, 24 h). **(B)** CCK-8 assay assessing LLC cell viability. **(C-D)** Flow cytometry analysis of apoptosis in LLC cells. **(E)** CCK-8 assay measuring T-cell viability. **(F)** Flow cytometry analysis of apoptosis in T cells. **(G)** Western blot analysis of PD-L1, CXCL10, and CXCL11 expression in co-cultures. **(H)** ELISA detecting CXCL10 and CXCL11 secretion in supernatants. **(I)** ELISA measuring IFN-γ and IL-2 levels in supernatants. **(J)** LDH release assay evaluating T-cell cytotoxicity. *n* = 3. The p-values in panels A-K were calculated using ANOVA with Tukey’s *post hoc* test. *p < 0.05 vs. Control. #p < 0.05 vs. T cells + si-NC. &p < 0.05 vs. T cells + si-CRTC1 + oe-NC.

Further investigation was conducted to determine whether the effects of tumor cell CRTC1 on T cell survival and cytotoxic capacity are mediated through the Notch1/Akt signaling pathway. A separate co-culture system was established, in which LLC cells transfected with plasmids knocking down CRTC1 (si-CRTC1) and overexpressing Notch1 (oe-Notch1) were co-cultured with CTLL-2 T cells, either unstimulated or activated with 100 U/mL IL-2. CRTC1 knockdown reduced the levels of CRTC1, Notch1, and p-Akt in cell lysates, while oe-Notch1 restored Notch1/p-Akt (**Figure 5A**). CRTC1 knockdown enhanced T cell-mediated LLC apoptosis and suppressed viability, whereas Notch1 overexpression reversed these effects (**Figures 5B–D**). Conversely, CRTC1 knockdown increased T cell viability and reduced apoptosis, which were counteracted by Notch1 overexpression (**Figures 5E–F**). CRTC1 knockdown downregulated PD-L1 and upregulated CXCL10/CXCL11 in cell lysates, alterations reversed by Notch1 overexpression (**Figure 5G**). Notch1 overexpression also reduced supernatant levels of IFN-γ, IL-2, CXCL10, CXCL11 (**Figures 5H–J**), as well as LDH release (**Figure 5K**). These results collectively confirm that CRTC1 regulates T cell activity via the Notch1/Akt pathway.

### 3.6 CRTC1 inhibition enhances the anti-tumor effects of PD-L1 blockade in xenografts via Notch1/Akt

**Figure 6:**
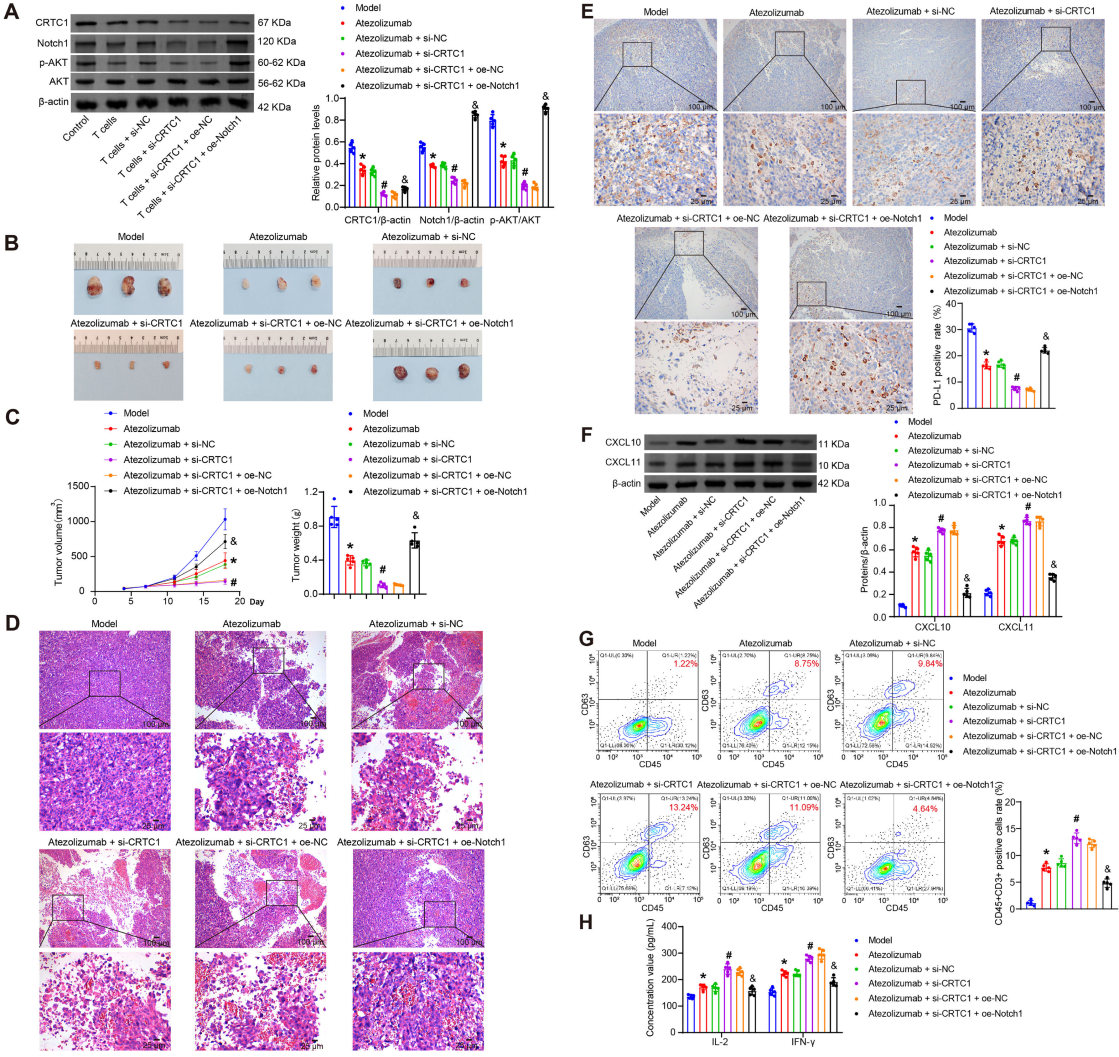
CRTC1 knockdown synergizes with PD-L1 blockade to suppress tumor growth *in vivo*. **(A)** Western blot analysis of CRTC1, Notch1, and p-Akt expression in Lewis xenograft tumors from C57BL/6J mice treated with Atezolizumab combined with plasmids knocking down CRTC1 (si-CRTC1) and overexpressing Notch1 (oe-Notch1) or their negative controls (si-NC or oe-NC). **(B)** Tumor size in mice bearing Lewis xenografts treated with Atezolizumab, CRTC1 knockdown, and Notch1 overexpression. **(C)** Tumor volume and weight of Lewis xenografts under combined treatment. **(D)** Representative H&E staining images of Lewis xenografts post-treatment. Scale bar = 100 μm (top) and 25 μm (bottom). **(E)** IHC analysis of PD-L1 expression in Lewis xenograft tumors. Scale bar = 100 μm (top) and 25 μm (bottom). **(F)** Western blot analysis of CXCL10 and CXCL11 expression in tumors. **(G)** Flow cytometry analysis of CD3+ T-cell infiltration in tumors. **(H)** ELISA measuring serum IFN-γ and IL-2 levels. *n* = 5. The p-values in panels **(A, C, E–H)** were calculated using ANOVA with Tukey’s *post hoc* test. *p < 0.05 vs. Model. #p < 0.05 vs. Atezolizumab + si-NC. &p < 0.05 vs. Atezolizumab + si-CRTC1 + oe-NC.

To interrogate the CRTC1-PD-L1 immune crosstalk *in vivo*, LLC cells with CRTC1 knockdown and Notch1 overexpression were subcutaneously injected into mice. Seven days after inoculation, atezolizumab was administered intraperitoneally. Combined CRTC1 knockdown and atezolizumab treatment amplified CRTC1/Notch1/p-Akt downregulation in tumors, while Notch1 overexpression restored Notch1/p-Akt (**Figure 6A**). CRTC1 knockdown enhanced atezolizumab’s anti-tumor effects, reducing tumor size, weight, and volume (**Figures 6B–C**) and increasing tumor cell death (**Figure 6D**). Notch1 overexpression reversed these benefits (**Figures 6B–D**). IHC results demonstrated that CRTC1 knockdown amplified the reduction in PD-L1 expression induced by atezolizumab, whereas Notch1 overexpression elevated PD-L1 expression in tumor tissues (**Figure 6E**). CRTC1 knockdown further elevated atezolizumab-induced CXCL10/CXCL11 secretion, suppressed by Notch1 overexpression (**Figure 6F**). Pearson correlation analysis revealed that CRTC1 exhibited positive correlations with PD-L1 and Notch1, respectively, while showing negative correlations with CXCL10/CXCL11 (**Supplementary Figures S1A**–D). Atezolizumab increased tumor-infiltrating T cells, augmented by CRTC1 knockdown but blocked by Notch1 overexpression (**Figure 6G**). Serum IFN-γ and IL-2 levels peaked in the combination group (Atezolizumab + si-CRTC1), while Notch1 overexpression reduced them (**Figure 6H**). These findings demonstrate that CRTC1 inhibition and PD-L1 blockade synergistically suppress tumor growth via Notch1/Akt signaling.

## 4 Discussion

Despite significant advancements, a substantial proportion of patients with advanced NSCLC exhibit limited clinical response rates to PD-L1 blockade therapy (30). Anti-PD-L1-based combination strategies hold greater promises for achieving favorable survival outcomes. Recent studies have identified that targeting tumor cell-specific biomarkers with inhibitors can effectively improve positive responses in preclinical models (31, 32). Atezolizumab (anti-PD-L1) has demonstrated efficacy across diverse therapeutic settings, including early-stage and metastatic NSCLC (33). Combination therapies may optimize the safety and efficacy of monotherapy while enhancing patient benefits. In this study, we identified candidate factors influencing atezolizumab efficacy. For the first time, we characterized CRTC1 as an oncogenic factor positively correlated with PD-L1. *In vitro* data revealed that CRTC1 in NSCLC cells upregulates PD-L1 by directly targeting the Notch1/Akt signaling pathway, thereby suppressing T-cell survival and cytotoxic activity, ultimately triggering tumor reprogramming in NSCLC cells. *In vivo* findings confirmed that CRTC1 reverses the therapeutic effects of atezolizumab in NSCLC via Notch1/Akt. We elucidated the regulatory mechanism of CRTC1-mediated immune evasion in NSCLC and propose that CRTC1-targeted inhibitors may serve as a potent therapeutic strategy to enhance patient sensitivity to anti-PD-L1 therapy.

Aberrant activation of CRTC family members is recognized as a critical driver of tumor progression (34). CRTC3 promotes melanocyte differentiation and tumorigenesis by integrating cAMP and MAPK/ERK signaling (35), while CRTC2 enhances autophagic flux via PI3K-AKT pathway activation, reducing paclitaxel sensitivity in ovarian cancer cells (36). CRTC1 forms fusion transcripts with mastermind-like 2 (MAML2) that function as transcriptional coactivators driving mucoepidermoid carcinoma development (37), including in lungs (38). CRTC dysregulation is closely associated with LKB1-mutant lung cancer pathogenesis (39). Specifically, CRTC2 accelerates LKB1-deficient lung cancer progression by stimulating ID1 (inhibitor of DNA binding 1) expression (40). CRTC1 promotes LKB1-deficient lung cancer progression through NEDD9 transcriptional induction (41) or glycosylated COX-2 protein activation (42). Here, we reveal a distinct mechanism by which CRTC1 influences wild-type NSCLC development via Notch1/Akt signaling. We demonstrate that CRTC1 overexpression drives NSCLC cell growth, while its knockdown suppresses proliferation.

Tumor cells subvert cytotoxic T-cell function through immune checkpoint ligand-receptor interactions (43). PD-L1-mediated evasion of T-cell surveillance represents a key biological mechanism of targeted immunosuppression (44). CRTC2 reportedly promotes hepatocellular carcinoma growth through Wnt/β-catenin pathway activation while downregulating the PD-L1/PD-1 axis, thereby enhancing immunotherapy resistance (16). In this study, we identify CRTC1 as a positive regulator of PD-L1 in NSCLC. We found that CRTC1 overexpression in tumor cells upregulates PD-L1, suppresses IFN-γ and IL-2 production, reduces CXCL10/11 secretion, and impairs T-cell cytotoxicity. CRTC1 knockdown potentiated the therapeutic efficacy of atezolizumab, evidenced by xenograft tumor regression, increased CXCL10/11 expression, and elevated serum concentrations of IFN-γ and IL-2. Tumor Tumor-infiltrating T lymphocytes, key effector cells of the immune system, can secrete anti-tumor cytokines (IFN-γ and IL-2). PD-L1 on cancer cells binds to PD-1 on antigen-stimulated T cells, triggering T-cell exhaustion and reducing IFN-γ and IL-2 secretion. IFN-γ induces intratumoral CXCL10/11 secretion, promoting CD8+ T-cell infiltration and enhancing antitumor immunity (45). Conversely, IFN-γ induces PD-L1 overexpression via JAK-STAT signaling activation, thereby compromising antitumor immunity (46). Notably, crosstalk between CRTC and JAK-STAT pathways has been documented to cooperatively regulate bone marrow homeostasis (47) and adipocyte differentiation (48). Additionally, aberrant activation of the PI3K/Akt/mTOR pathway in NSCLC enhances PD-L1 protein translation, while PD-L1 overexpression reciprocally activates PI3K/Akt/mTOR signaling (49). Our findings demonstrated that CRTC1 stimulates Akt phosphorylation to induce PD-L1 activation. During tumorigenesis, Akt stimulates CREB activation and downstream gene expression in a phosphorylation-dependent manner (50). Anti-PD-L1 therapy may initiate negative feedback loops through suppression of IFN-γ or Akt pathways—a phenomenon warranting further investigation. These findings collectively indicate that CRTC1 regulates immunotherapy for NSCLC through the PD-L1/PD-1 axis.

As a multifunctional transcriptional coactivator, systemic inhibition of CRTC1 does carry inherent risks of off-target effects, especially in metabolic and developmental contexts. CRTC1 deficiency may contribute to insulin resistance and obesity by dysregulating glucose and lipid metabolism (51, 52). CRTC1 is recognized as a modulator of cerebral metabolism, with potential implications for disrupting fetal development and postnatal neurodevelopment (53). Furthermore, CRTC1 depletion reduces hippocampal glucose metabolic capacity and drives depression-like behaviors in mice (54). These risks primarily correlate with systemic exposure. To advance clinical translation while minimizing off-target risks, future research warrants comprehensive investigation into developing highly selective inhibitors, achieving tumor-targeted delivery systems, and conducting rigorous preclinical and clinical safety assessments.

Notch1 acts as an oncogenic driver by promoting NSCLC cell invasion, metastasis, and malignant transformation through context-dependent protein interactions (55). Canonical Notch signaling involves ligand binding and γ-secretase-mediated proteolytic cleavage to generate the Notch intracellular domain (NICD), which translocates to the nucleus and binds nuclear factors to regulate transcriptional activity of downstream targets (56). Foundational studies indicate that the stability and activity of nuclear NICD1 are modulated by interaction partners, facilitating NSCLC proliferation, metastasis, and stemness maintenance (57, 58). Furthermore, CRTC1 is an established transcriptional coactivator of CREB (13), while downstream effectors of the Notch pathway rely on transcriptional regulation (59, 60). Notably, potential crosstalk between the Notch pathway and CREB activity has been documented (61). Our work demonstrated that CRTC1 interacts with Notch1 and positively regulates the Notch1/Akt signaling axis, thereby driving NSCLC cell growth.

Tumor immunity is similarly modulated by the Notch signaling pathway. Notch1 upregulates PD-1 expression on T cells, suppresses T-cell proliferation and activation, and impairs antitumor immune responses (62). Notch1 ablation enhances cancer cell responsiveness to PD-1/PD-L1 blockade (63). NSCLC patients with hypermutated Notch, leading to functional inactivation, exhibit improved prognosis following PD-1/PD-L1 blockade therapy (28, 64). These findings highlight Notch as a predictive biomarker for immunotherapy in NSCLC. In this study, we revealed that CRTC1 knockdown amplifies anti-PD-L1 tumor immunity via the Notch1/Akt pathway, improving atezolizumab efficacy and response rates. Thus, targeting CRTC1 and Notch signaling may represent a potential strategy to enhance NSCLC sensitivity to immunotherapy.

This study has several limitations. Inclusion of an atezolizumab-treated clinical cohort could further clarify the roles of CRTC1 and Notch signaling. The absence of CRTC1/Notch conditional knockout mice limits our ability to dissect NSCLC tumor-induced immunosuppression. Although we demonstrated CRTC1/Notch1 interaction in NSCLC, the mechanism by which CRTC1 activates Notch/Akt signaling requires further investigation. Whether CRTC1 activates the Notch1/Akt pathway through integrating scaffolding functions with transcriptional regulatory capacities warrants further investigation. Future studies exploring CRTC1/Notch pathway involvement in anti-PD-1/PD-L1 therapy resistance or toxicity will deepen understanding of combination therapies.

In conclusion, we identified CRTC1 as a promoter of tumor immune evasion and cancer progression in NSCLC via the Notch/Akt pathway. Suppression of CRTC1 expression in NSCLC cells may serve as a viable strategy to potentiate anti-PD-1/PD-L1 immunotherapy.

## Data Availability

The raw data supporting the conclusions of this article will be made available by the authors, without undue reservation.

## Supplementary material

Supplementary Figure 1 Pearson correlation analysis of the correlations between CRTC1 and PD-L1 **(A)**, Notch1 **(B)**, CXCL10 **(C)**, and CXCL11 **(D)** in the tumor tissues of mice.
