# Supplementary figures and images for: CRTC1 enhances PD-L1-mediated tumor immunosuppression in non-small cell lung cancer via the Notch1/Akt signaling pathway
Source: Front Immunol. 2025 Sep 5;16:1658679. doi: 10.3389/fimmu.2025.1658679 (PMC12446252; doi:10.3389/fimmu.2025.1658679)

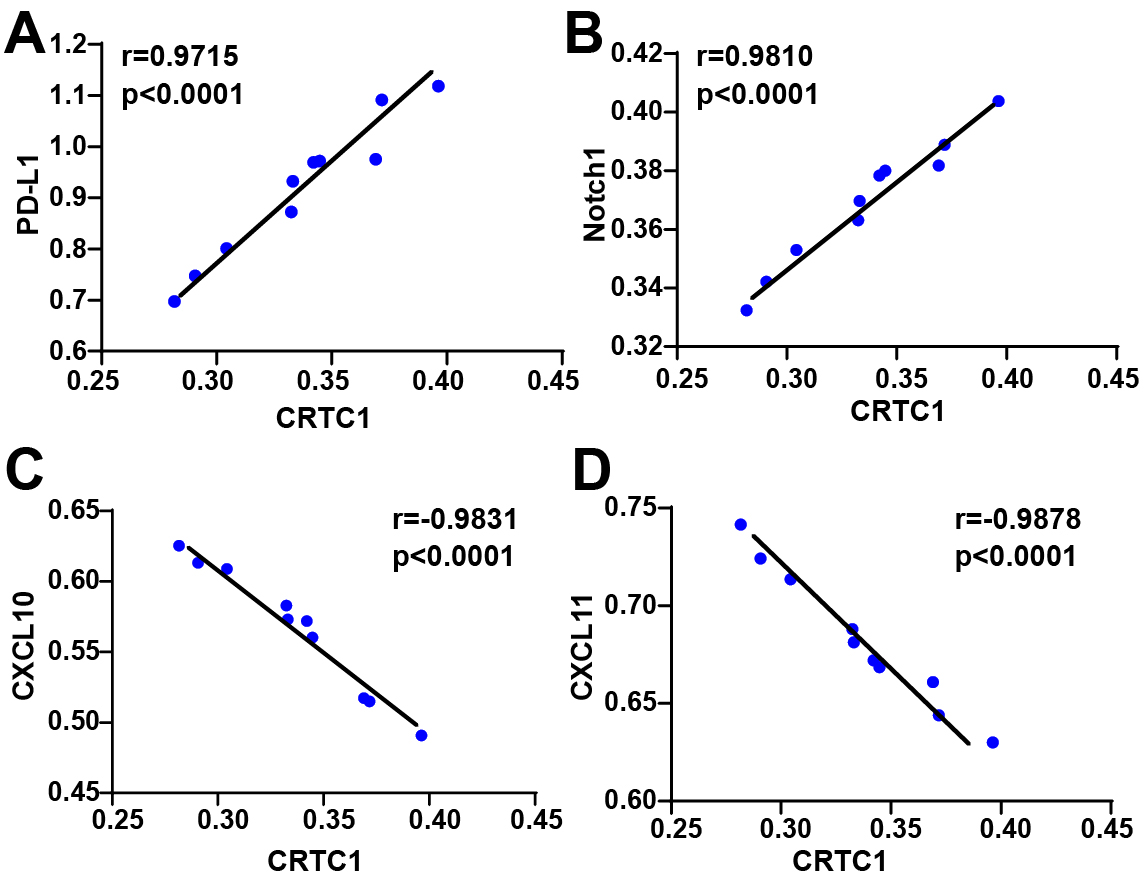

Supplement: Supplementary Figure 1 — Pearson correlation analysis of the correlations between CRTC1 and PD-L1 (A), Notch1 (B), CXCL10 (C), and CXCL11 (D) in the tumor tissues of mice. [file Image1.jpeg]
