# Supplementary material for: CRTC1 enhances PD-L1-mediated tumor immunosuppression in non-small cell lung cancer via the Notch1/Akt signaling pathway
Source: Front Immunol. 2025 Sep 5;16:1658679. doi: 10.3389/fimmu.2025.1658679 (PMC12446252; doi:10.3389/fimmu.2025.1658679)

**Figure 1G**

CRTC1

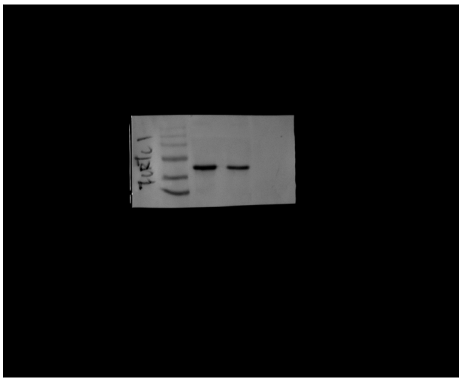

β-actin

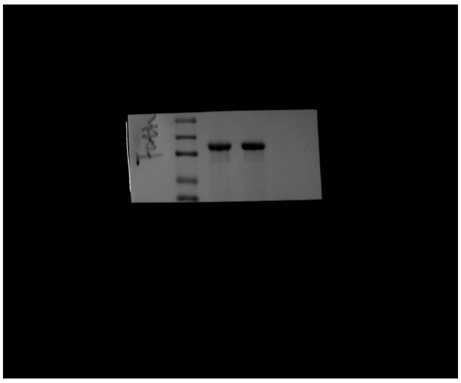

**Figure 1H**

CRTC1

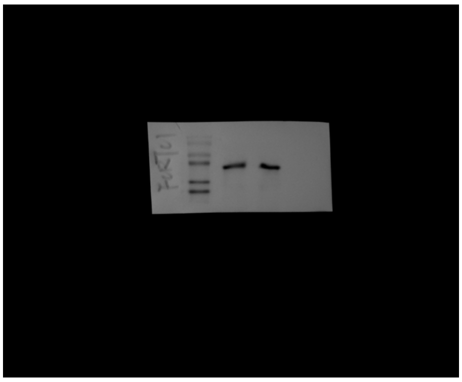

β-actin

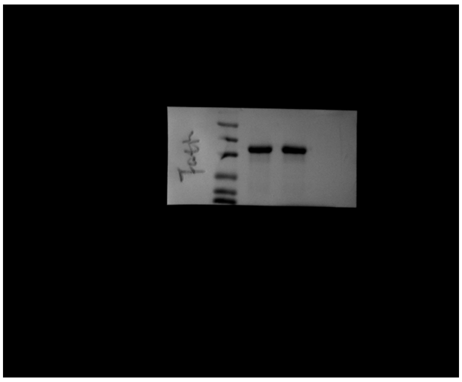

**Figure 2A**

**Human**

CRTC1

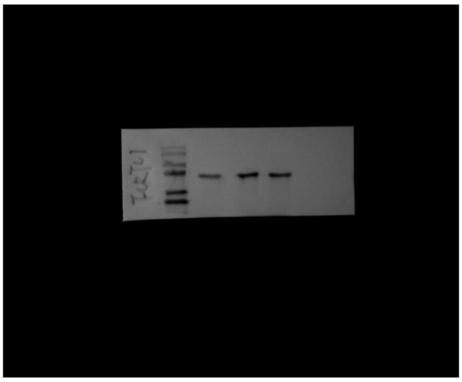

β-actin

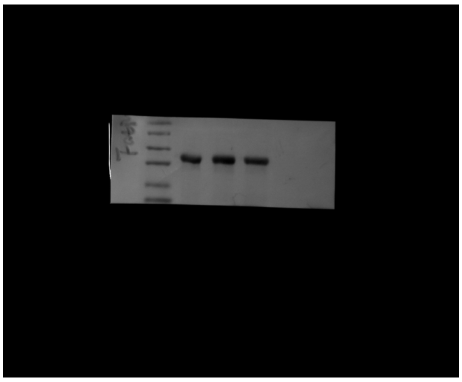

**Mouse**

CRTC1

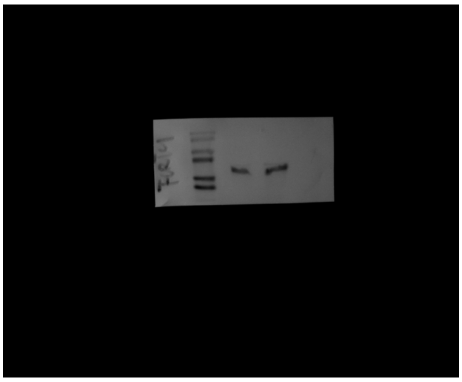

β-actin

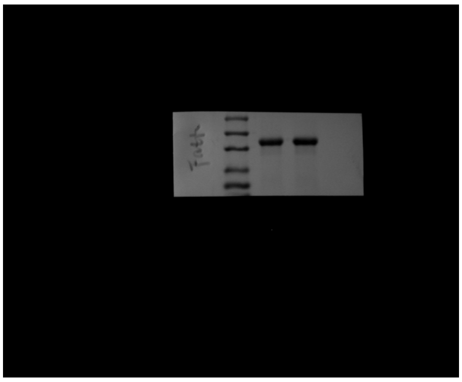

**Figure 2B**

**A549**

CRTC1

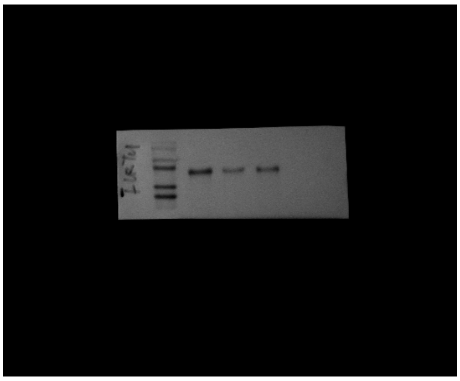

β-actin

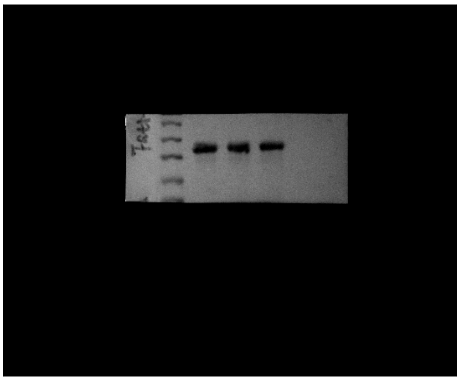

**NCI-H1299**

CRTC1

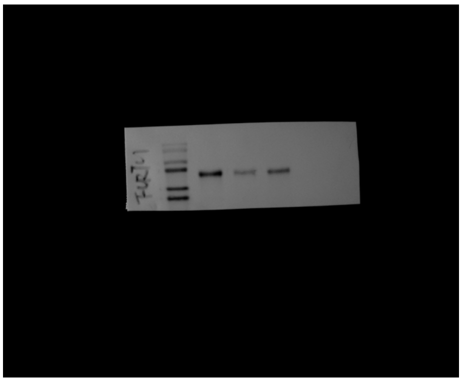

β-actin

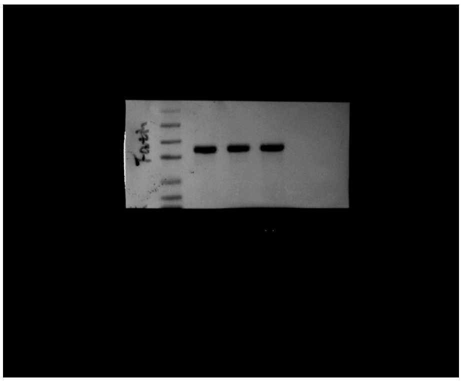

**Figure 2C**

**A549**

CRTC1

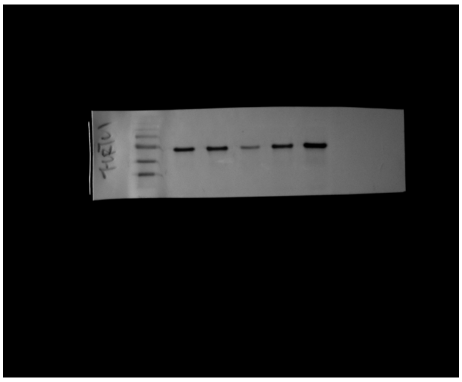

β-actin

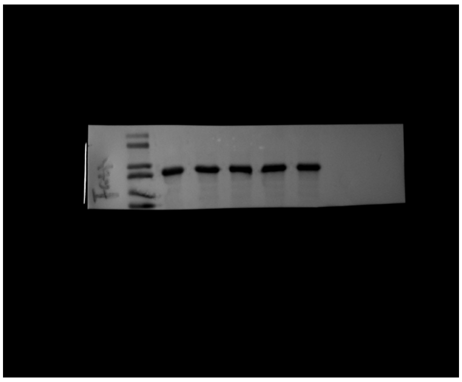

**NCI-H1299**

CRTC1

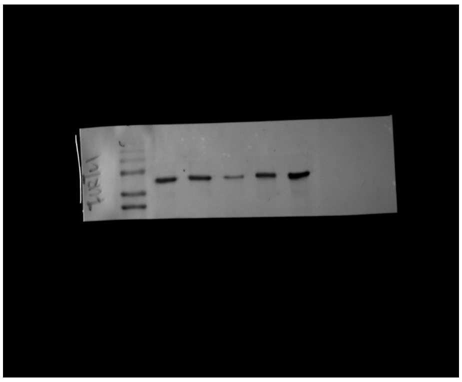

β-actin

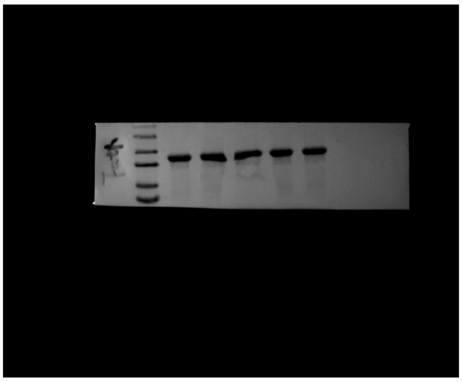

**Figure 2I**

**A549**

PD-L1

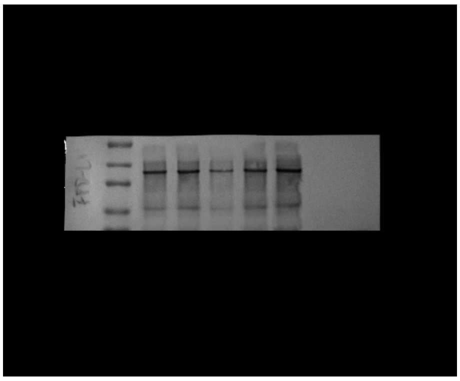

β-actin

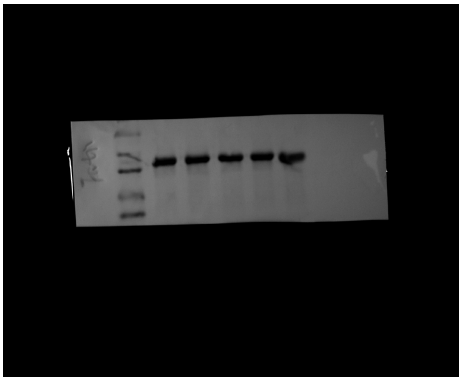

**NCI-H1299**

PD-L1

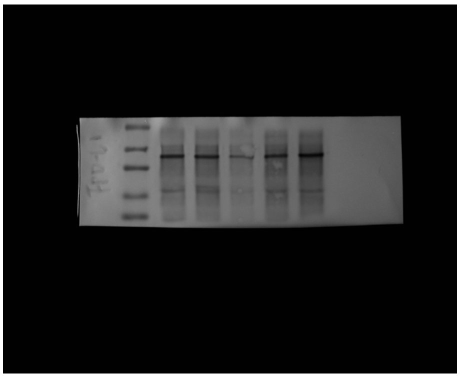

β-actin

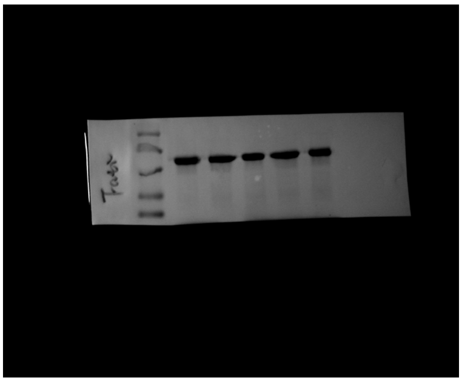

**Figure 3A**

**A549**

Notch1

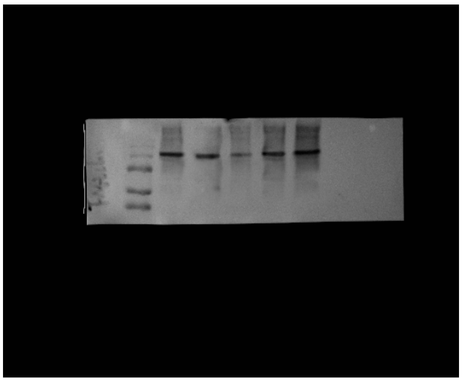

p-AKT

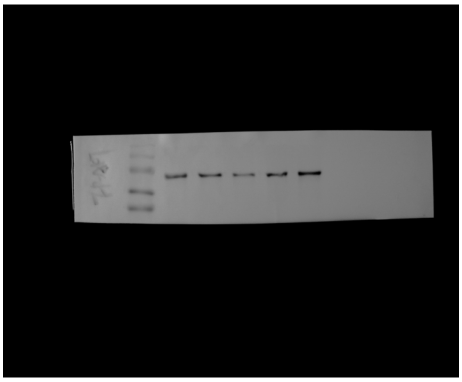

AKT

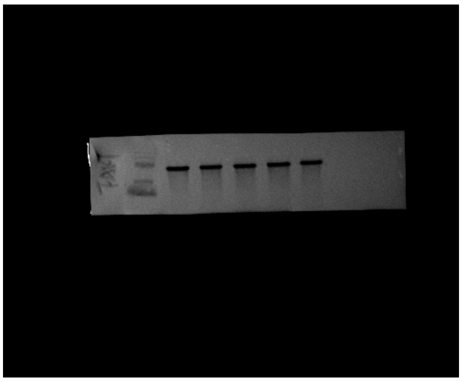

β-actin

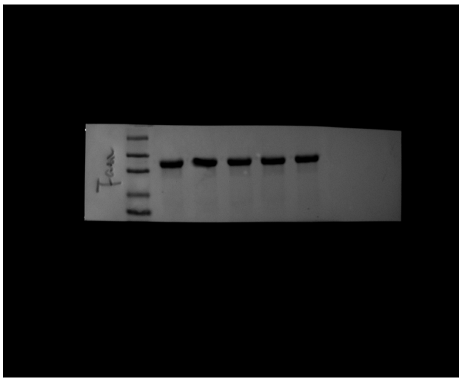

**NCI-H1299**

Notch1

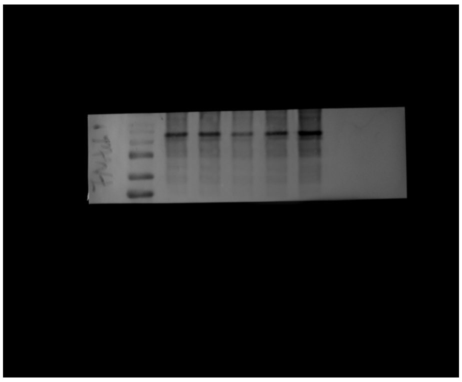

p-AKT

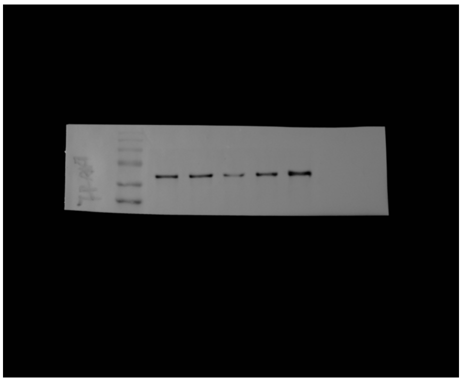

AKT

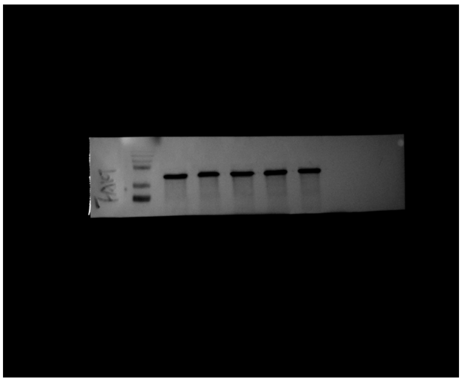

β-actin

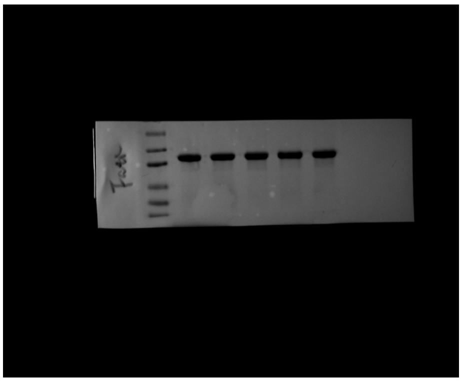

**Figure 3C**

**NCI-H1299**

CRTC1

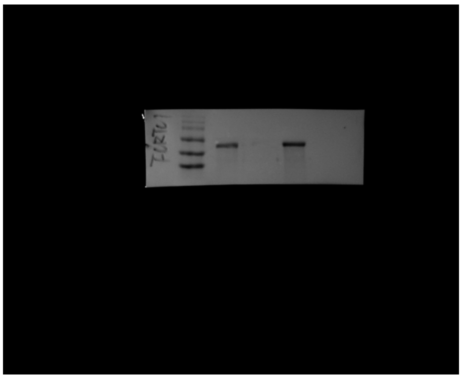

Notch1

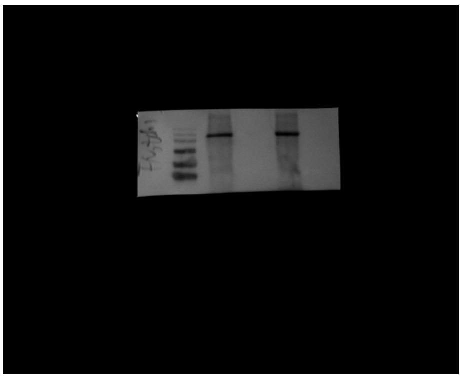

**A549**

CRTC1

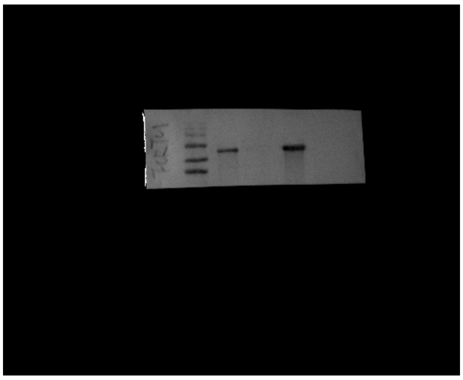

Notch1

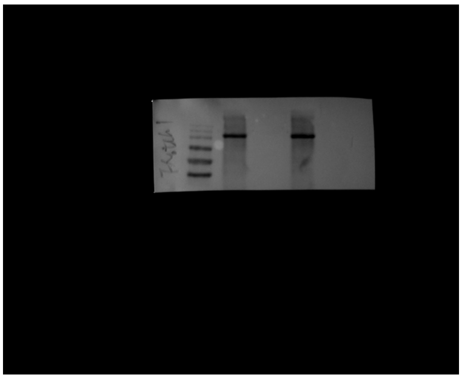

**NCI-H1299**

CRTC1

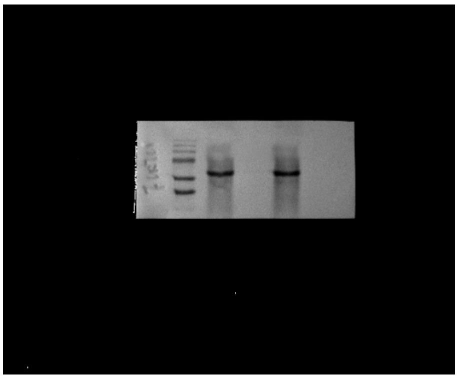

Notch1

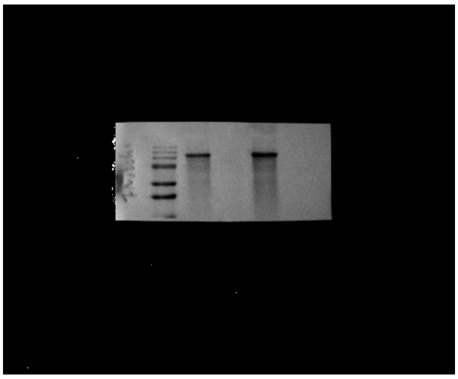

**A549**

CRTC1

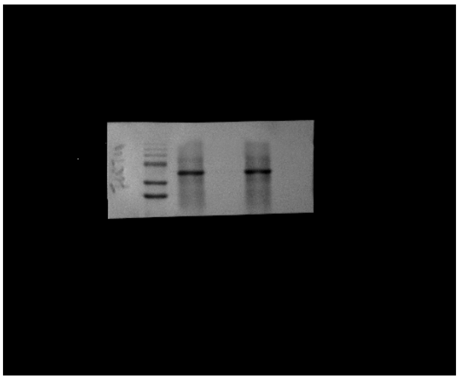

Notch1

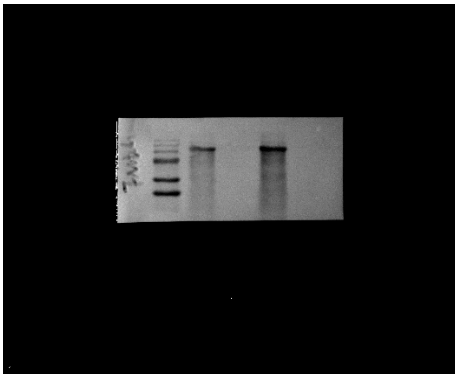

**Figure 3D**

**A549**

CRTC1

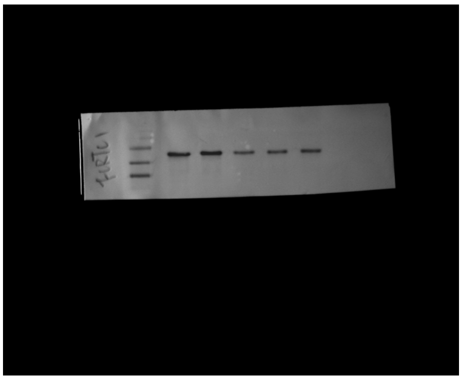

Notch1

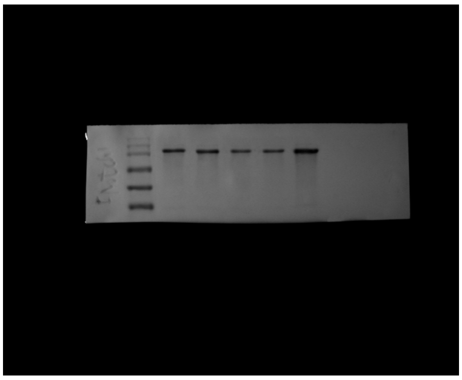

p-AKT

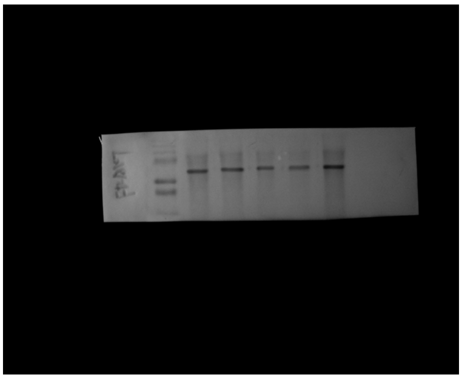

AKT

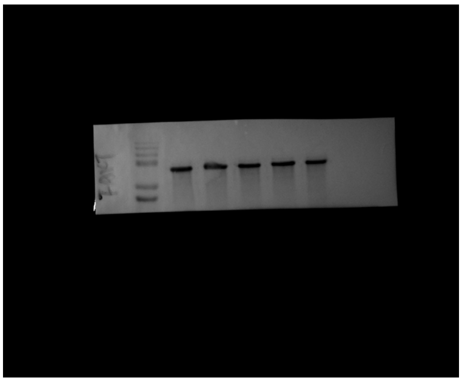

β-actin

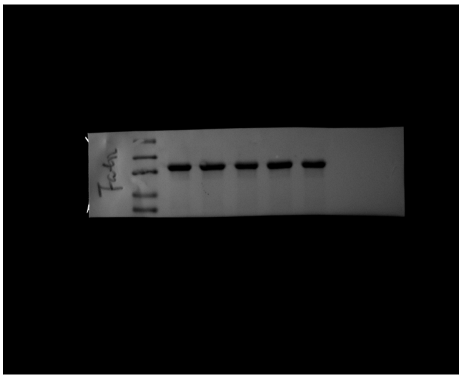

**NCI-H1299**

CRTC1

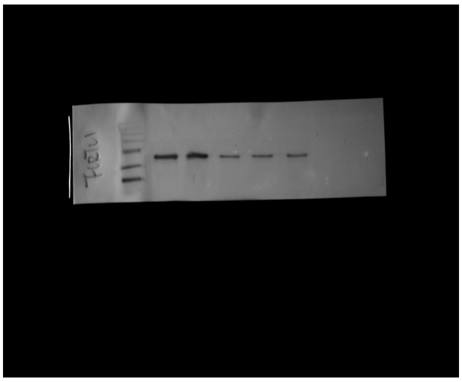

Notch1

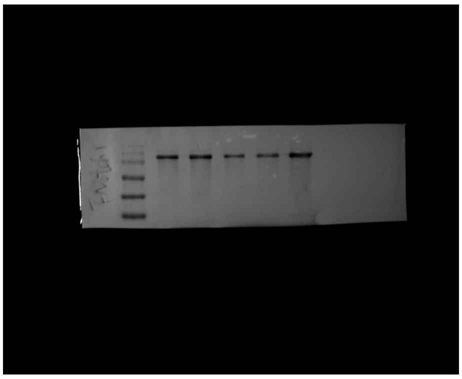

p-AKT

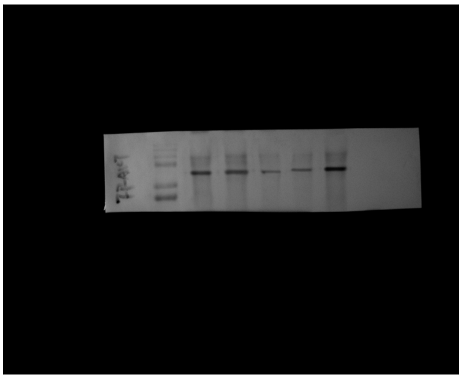

AKT

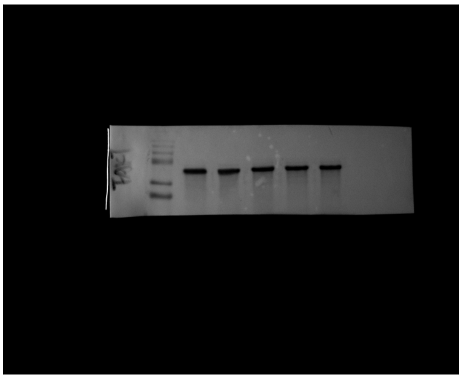

β-actin

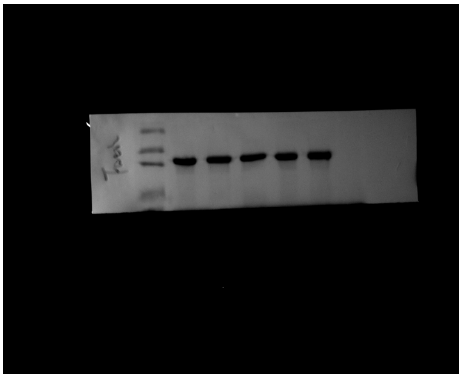

**Figure 3I**

**A549**

PD-L1

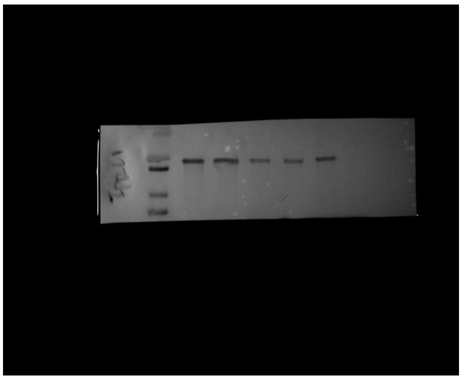

β-actin

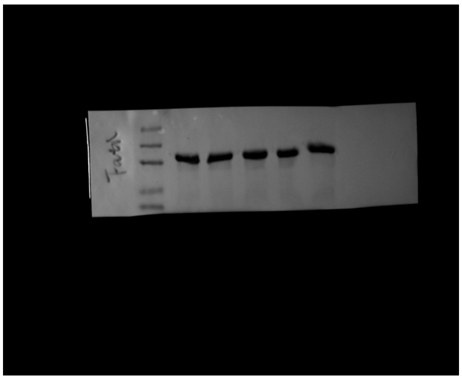

**NCI-H1299**

PD-L1

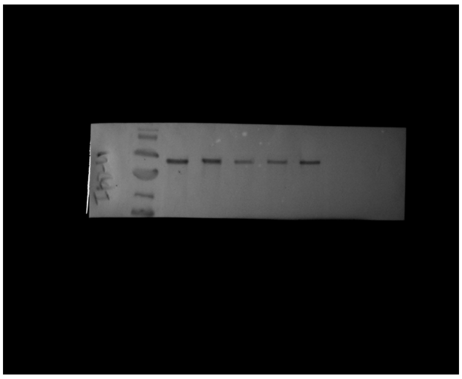

β-actin

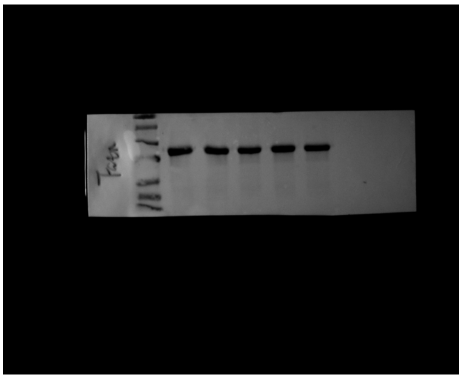

**Figure 4A**

CRTC1

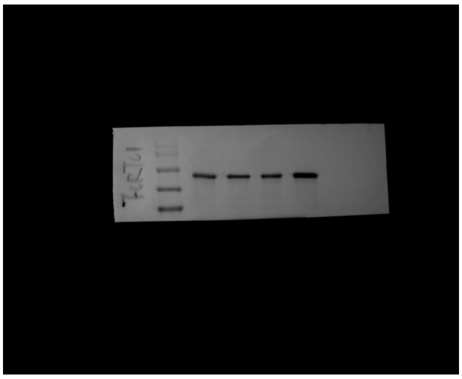

β-actin

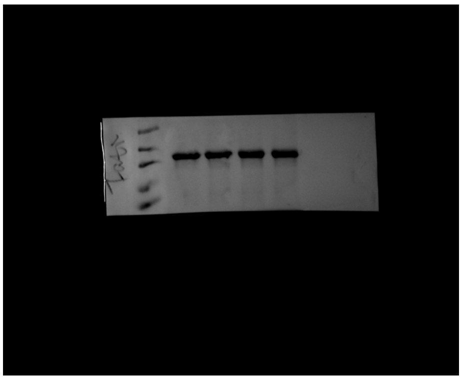

**Figure 4G**

PD-L1

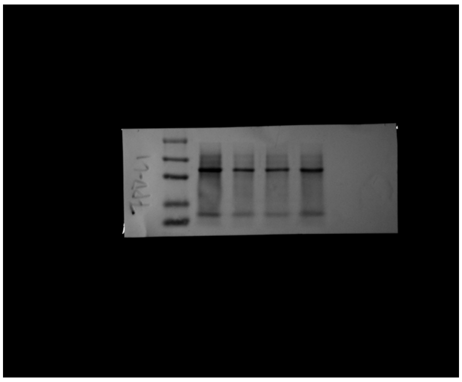

CXCL10

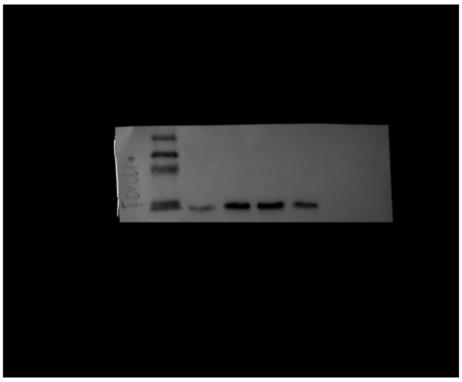

CXCL11

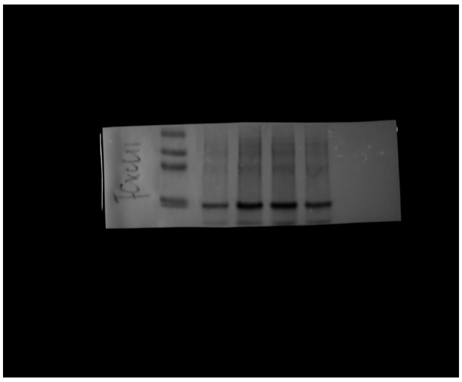

β-actin

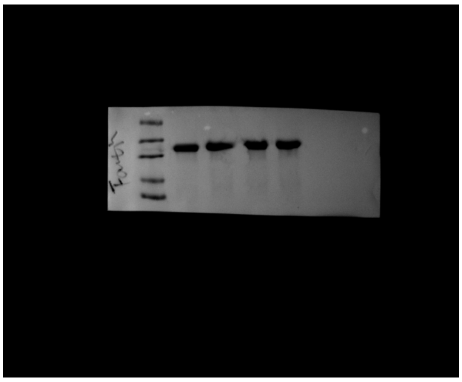

**Figure 5A**

CRTC1

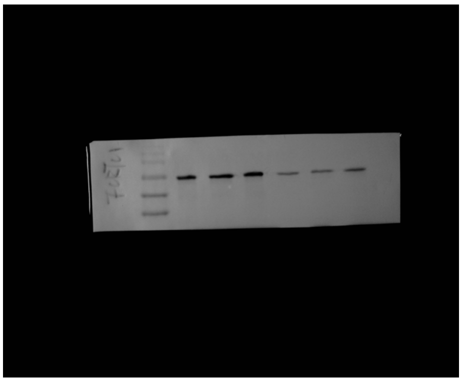

Notch1

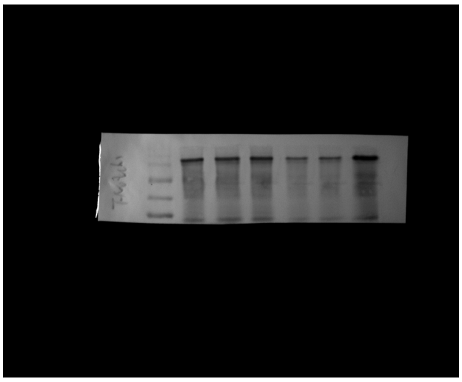

p-AKT

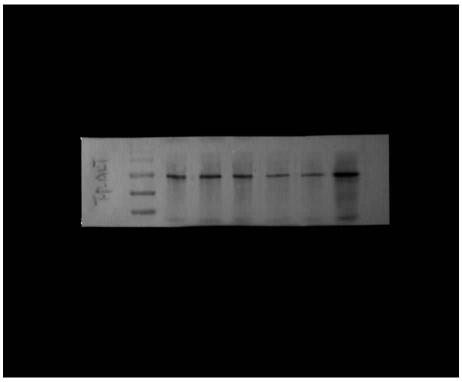

AKT

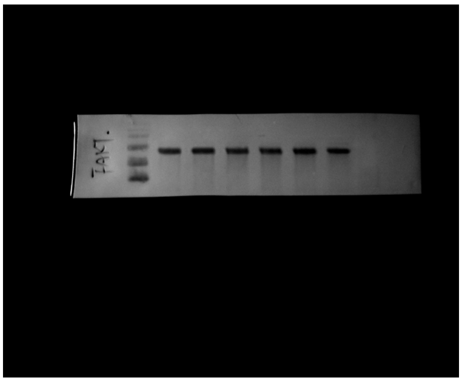

β-actin

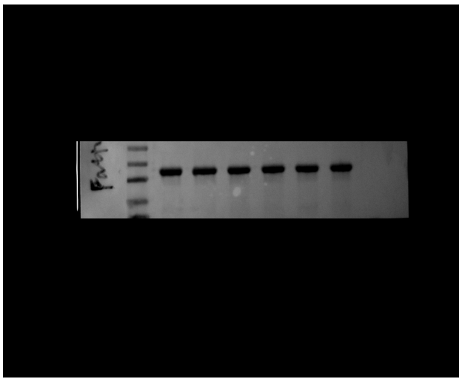

**Figure 5G**

PD-L1

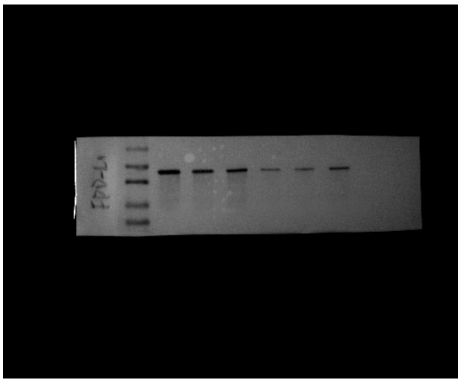

CXCL10

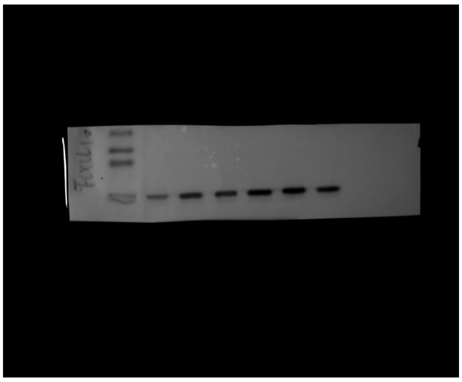

CXCL11

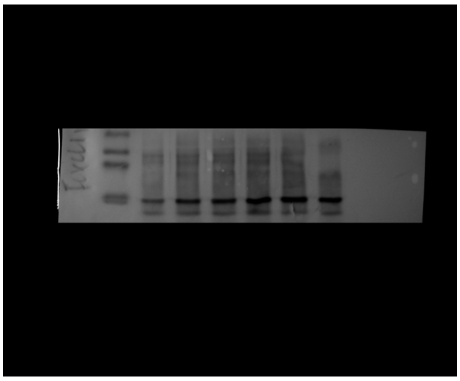

β-actin

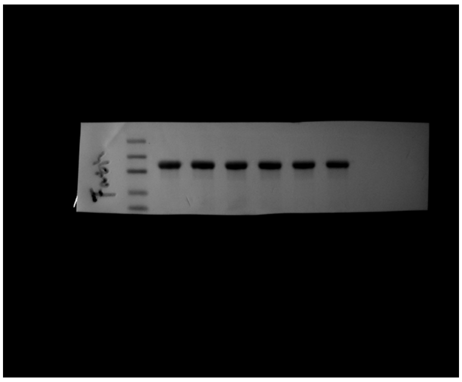

**Figure 6A**

CRTC1

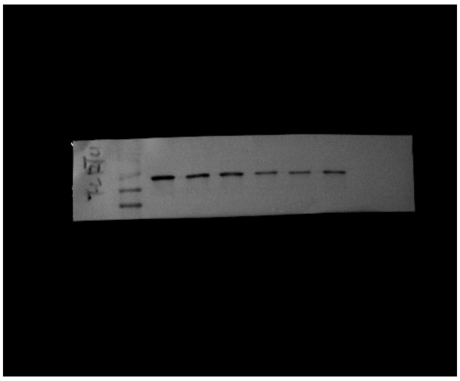

Notch1

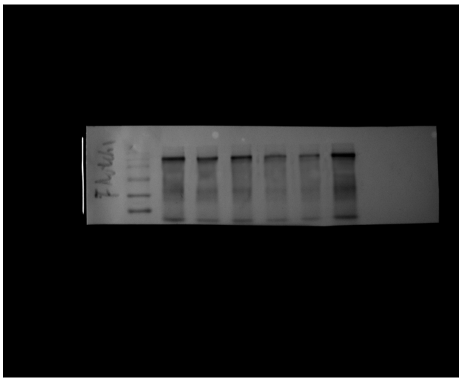

p-AKT

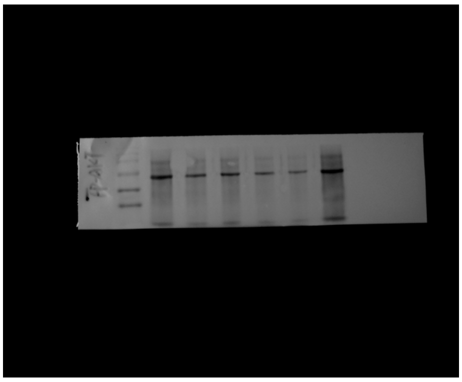

AKT

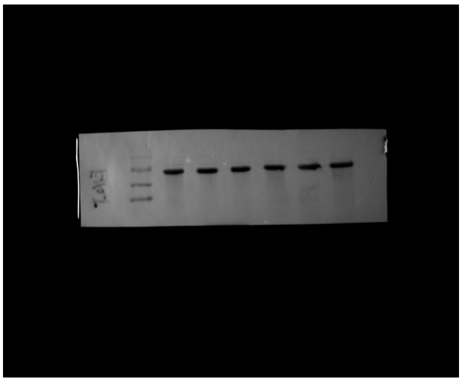

β-actin

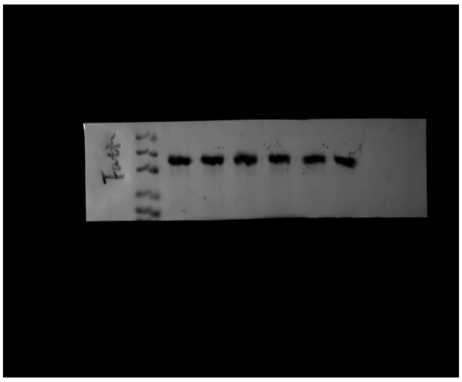

**Figure 6F**

CXCL10

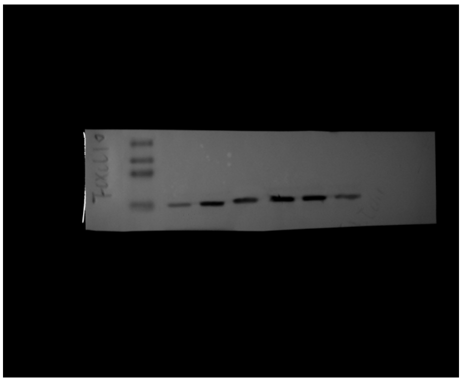

CXCL11

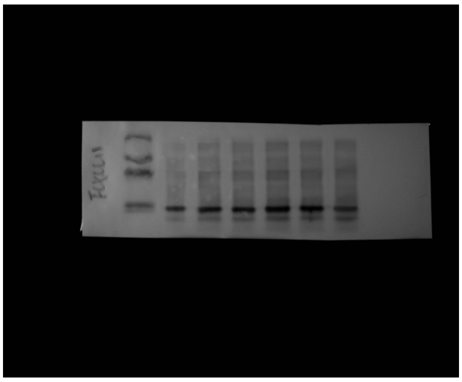

β-actin

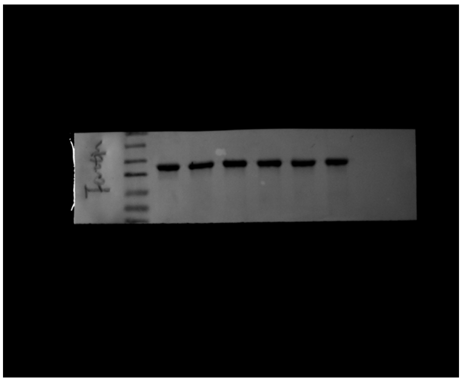

Supplement: Supplementary file 2 [file DataSheet1.pdf]
